# Ecological cooperative merging control of heterogeneous electric vehicle platoons

**DOI:** 10.1371/journal.pone.0309930

**Published:** 2024-11-12

**Authors:** Tian Luo, Xiaobin Liu

**Affiliations:** School of Automotive Engineering, Lanzhou Institute of Technology, Lanzhou, China; Southwest Jiaotong University, CHINA

## Abstract

Vehicle platooning improves energy savings via vehicle-to-vehicle (V2V) communication. Ecological cooperative adaptive cruise control (Eco-CACC) is implemented in platoons for merging task by using regrouped platoon models. The merging positions are selected in the middle and tail of an original platoon with a two-vehicle sub-platoon. The distributed nonlinear model predictive controller based on signal temporal logic (DNMPC-STL) approach is developed to model the Eco-CACC merging strategy. The performance of the Eco-CACC merging strategy is modeled by objective control for a predecessor-leader following (PLF) topology. The results demonstrate that merging positions located in the tail exhibit superior performance and can be used to improve stability, tracking performance, energy consumption efficiency and SOC of battery.

## Introduction

Recent developments in connected and automated vehicles (CAVs) have increased the need for eco-driving and autonomous driving. Automated connected vehicle technology can be applied mainly to adaptive cruise control (ACC) [1, 2], Eco-ACC [3], cooperative adaptive cruise control (CACC) [4], and Eco-CACC [5, 6]. The Eco-CACC system is one of the most important technologies used in advanced driving assistant system (ADAS). The CAVs communicate and collaborate with each other in a platoon. This process yields energy savings because following vehicles experience less air drag and cover less distance. Moreover, ecological driving in different traffic environments can be achieved. Researchers studying platoons have focused mainly on string stability and the tracking performance [7–9], which are the basis of homogeneous and heterogeneous platoons.

Recent research has emphasized that the application of CAV technology in heterogeneous platoons increases energy savings, SOC of battery, safety performance, stability and tracking performance. Therefore, such technologies have garnered widespread interest. Moreover, it is very important to apply CAV technology in heterogeneous platoons to evaluate Eco-CACC in real mixed traffic scenarios.

### Heterogeneous platoon model

Two basic vehicle dynamics models are currently being adopted for heterogeneous platoons: the linear platoon model and the nonlinear platoon model.

Linear models can be categorized into model predictive controller (MPC) [10] and proportional-integral-differential (PID) [11, 12] controller. Based on these approaches, the application of a linear platoon, coupled with state fluctuations and applicable platoon sequencing, contributes to achieving safety, stability, and predictability objectives. Additionally, placing restrictions on the leading car’s velocity overshoot helps maintain smooth and safe platoon dynamics [13]. An Eco-CACC strategy appears to addresses the complexities of managing heterogeneous platoons by combining distributed linear feedback control for following vehicles and model predictive control for the leading vehicle. The reported fuel savings in simulations indicate the strategy’s potential benefits in terms of fuel economy, riding comfort and driving efficiency under different velocity ranges [14]. A new vehicular platoon dynamics model focuses on developing distributed linear control protocols for CACC, emphasizing a constant spacing policy. The analysis involves leveraging algebraic graph theory and the Routh-Hurwitz (R-H) stability criterion to establish necessary and sufficient conditions for stability across various network topologies. This approach aims at providing a robust and widely applicable solution for managing vehicular platoons in diverse settings [15].

It is a widely held view that vehicle dynamics can be represented as nonlinear models in the real world. The implementation of this approach involves an adaptive tube-based nonlinear model predictive control (AT-NMPC) approach for designing autonomous cruise control systems and employing two separate models to define a constrained receding horizon optimal control problem. The aim of this strategy is to enhance adaptability and performance while considering uncertainties in the control process [16]. Then, the distributed reference governor approach is tailored for vehicle platoons with CACC. The method is designed to ensure string stability, handle platooning constraints, and improve energy economy of the platoon. The use of high-fidelity models and evaluations help validate the effectiveness of the proposed approach in a realistic simulation environment [17]. A real-time NMPC framework for an Eco-CACC system is proposed, emphasizing substantial fuel savings through a combination of optimal eco-driving and ACC principles. The implementation of electric vehicles underscores the consideration of unique characteristics and efficiency considerations associated with electric power-train [18]. The effectiveness of the Eco-CACC approach was evaluated for an electric vehicle in a platoon setting. Considering various speed profiles and the balance between inter-vehicular distance reduction and velocity profile smoothing, a comprehensive approach to achieving the energy efficiency and ecological driving is needed [19]. Multi-objective optimal control is employed by the DMPC in an electric vehicle platoon. This strategy is complemented by a braking force distribution approach for the following vehicles in the platoon, with the ultimate objective of achieving economical driving for the entire platoon [20]. By combining eco-driving principles, vehicle tracking, and the predictive capabilities of nonlinear MPC, the Eco-CACC strategy aims at providing a comprehensive solution for minimizing energy consumption in autonomous vehicle platoons. The collaborative nature of the control strategy enables the platoon to operate in a coordinated manner, further enhancing energy efficiency [21].

### Heterogeneous platoon communication topology

Communication errors and delays can be amplified as they travel along with the platoon. It has commonly been acknowledged that a good communication topology improves platoon stability, prevents non-rear collisions and increases the energy savings of vehicle platoons.

The advantages of connected and automated vehicle systems include the formation of platoons, where vehicles are tracked based on a shared speed profile generated by the leading vehicle. Various communication topology and V2V communication methods are employed to facilitate data exchange among platoon vehicles, ensuring the integrity of information flow within the platoon. Previous research has shown that predecessor-follower (PF) communication topology has difficulty ensuring platoon stability. It is obvious for PF that the following vehicle influences the preceding vehicle only [22, 23]. Such treatment in [24], reference discusses a treatment for a platoon control, focusing on distributed receding horizon control algorithms and stability conditions. The treatment is specifically applicable to limited communication topology (PF and PLF) and addresses stability under speed fluctuations in the platoon. The distinction between PF and LF string stability, along with the associated communication, is also highlighted. The deployment of vehicle-to-vehicle communication technologies has led to the emergence of various communication topology such as TPF and multiple-PF styles [25–27]. The diverse range of topology presents new challenges for the platoon control, emphasizing the need for a systematic and integrated approach to address the complexities associated with different communication structures. According to analysis, PLF allows for information exchange with both the leading and predecessor vehicles, contributing to stability, while maintaining a lower computational burden compared to two-predecessor-leader following (TPLF) topology [28]. Therefore, our research emphasizes the advantages of the PLF communication topology in terms of platoon stability and computational efficiency.

### Stability of merging strategy on Eco-CACC

The mentioned platooning strategies are designed for a CAV environment, with a specific emphasis on highway maneuvers. The foundation was proposed in 1993 for achieving safe and efficient platoon maneuvers, including merges, splits, and lane changes. These strategies contribute to the overall optimization of traffic flow and coordination within a connected and autonomous vehicle platoon on highways [29].

For a merging task of sub-platoon, the position that needs to be merged is determined based on high-level decisions with regards to the design. Platoon merging is not only about forming longer platoons but also has specific objectives related to reducing information exchange errors and minimizing impacts on the existing vehicles [30]. The strategy of merging at the tail for one vehicle, under the decision of the platoon leader, helps achieve these goals [31]. The overall process is designed to enhance the efficiency and safety of CAV platooning systems.

Generally, the leader serves as a central hub for information exchange and plays a critical role in optimizing the merging position for the sub-platoon. An approach described involves allowing merging in the middle to increase platoon flexibility while keeping the platoon leader unchanged. The overall implementation results in benefits such as string stability, energy savings, and taking into account the schedules and destinations of all vehicles in the regrouped platoon [32].

A set of studies was conducted to consider single vehicle merging to a platoon. An Eco-ACC system that utilizes a control terminal set for a single vehicle and its interaction with the vehicle ahead. The emphasis is on fuel economy, achieved by avoiding unnecessary braking and optimizing the control strategy for smoother speed adjustments. This style of ACC system aligns with the broader goal of making driving more environmentally friendly and fuel-efficient [33]. Some researchers focused on sub-platoons that consist of two vehicles that merge into a platoon. A switched pinning control algorithm triggered by events was proposed within a multi-agent system. The algorithm focuses on platoon regrouping, and the pinning agents receive target velocities from external devices. Notably, the approach considers the computational cost of MPC in the steady state, suggesting an emphasis on efficiency in the control strategy [34]. Thus, in other studies, the strategies proposed in references [35–37] involves the simultaneous operation of multiple vehicles, with a focus on sub-platoon planning for merging clusters of vehicles into a platoon on a single-lane highway. A rolling horizon-based systematic trajectory planning (RSTP) approach for merging two CAV platoons was developed at a mainline-ramp intersection. The trajectory planning is optimized using a mixed integer nonlinear program (MINLP) and involves well-designed trajectory control zone and pre-merging subzone to ensure safe and smooth merging [38]. Some researchers focused on sub-platoons that consist of two vehicles or multiple vehicles that merge into an original platoon.

Therefore, it is necessary to review the state-of-the-art in CAV platoon operations and conclude the internal and string stability. Stabling and merging are two basic operations in vehicle platooning [30]. It is key for keeping platoon stability to maintain the desired gap and speed. Constant distance and constant time headway are commonly used to guarantee individual vehicle stability [39].

The string stability is achieved by stability strategy in a platoon [8, 40]. To prove string stability for merging task in a heterogeneous platoon, two methods widely can be utilized. These methods accommodate the diverse characteristics and behaviors of the vehicles in the platoon: frequency domain analysis method and Lyapunov method.

For frequency domain analysis methods, the transfer functions of different vehicles and solve the overall system’s frequency characteristics. A low pass filter with unity gain on Eco-CACC structure is proposed [41]. The string stability transfer function is expressed by the spacing policy, the feedback controller and the feedforward controller. The string stability of vehicles equipped with Eco-CACC in a platoon is expressed by the transfer function as magnitude frequency. To shorten following gap and enable the following vehicle in the sub-platoon to complete the merging task, a following-merging rule is proposed. The disturbance transfer function is used to keep the stability of the mixed traffic flow formed by merging vehicle platoons [42]. A new policy considering the side-vehicle merging behavior of connected cruise control (CCC) that is frequently happening at the high-speed platoon is proposed. By designing and applying the nonlinear range policy, the driving safety and comfort can be significantly improved when the sub-platoon merging behavior suddenly occurs. The string stability analysis is performed according to the head-to-tail transfer function [43].

For the Lyapunov method. This method is suitable for nonlinear systems and can handle vehicle heterogeneity. Hierarchical CAV merging control framework of a two-layer design is proposed [44]. The local stability is widely proved by the Lyapunov local stability and asymptotic local stability.

### Contributions

In the paper, a comprehensive analysis of Eco-CACC merging strategy is conducted to fill the gap. We focused on sub-platoons that consist of two vehicles or multiple vehicles that merge into an original platoon. Two merging positions strategy of CACC and SOC of battery for a heterogeneous platoon are evaluated. Therefore, three main contributions are highlighted to set our work apart from existing methods: the air drag coefficient is considered to be modified due to the different distance between predecessor and followers for PLF topology. According to the drag correction factor model, the reduction efficiency of aerodynamic drag is evaluated to inflect following compactness in a platoon; The SOC of battery and demand power for two merging positions are quantitatively investigated; It is established for PLF topology with DNMPC-STL approach to evaluate energy consumption, string stability, the driving stability and SOC of battery.

### Article structure

This paper is organized as follows: In the Methods section, a dynamic model of a battery electric vehicle is introduced, and the drag correction factor is modified. The nonlinear longitudinal dynamics platooning model is modeled based on DNMPC. Further extensions of DNMPC-STL to dynamic platooning and multi-objective platooning with leaders and predecessor followers are proposed. In the Analysis section, the merging positions are selected in the middle (style 1) and at the tail (style 2) of the original platoon, and the Eco-CACC merging strategy is applied to evaluate the stability, tracking performance and the driving stability of the reorganized platoon. The performance results are given in the Results section for two styles of experiments. Finally, in the Conclusion section, this paper is concluded, and future research directions are provided.

## Methods

### Heterogeneous platoon model

#### Vehicle dynamics model

According to the vehicle longitudinal dynamics, five forces are needed for electric vehicle movement considering road slopes. The rolling resistance (*F*_*f*_) is a force caused by the nonelastic effects that resist the driving when vehicles are rolling on the surface [45],

$$F_{f}=mg\;\cos\;\alpha$$ (1)

The aerodynamic drag (*F*_*w*_) is a force that acts opposite to the driving direction [8] as,

$$F_{w}=\frac{1}{2}C_{D}A_{e}\rho v^{2}$$ (2)

The uphill grade resistance (*F*_*i*_) is the resistance that acts parallel to the road surface [8].


$$F_{i}=mg\;\sin\;\alpha$$ (3)

Generally, the rotational acceleration force is approximated 5% of the linear acceleration force [45]. The linear acceleration force (*F*_*j*_) and rotational acceleration force (*F*_*a*_) are the forces vehicles to drive forward as,

$$F_{j}+F_{a}=1.05m\frac{dv}{dt}$$ (4)

where *m* represents the vehicle mass; *g* is the gravity acceleration; *f* is the coefficient of rolling resistance; *ρ* is the air density; *C*_*D*_ is the air drag coefficient; *A*_*e*_ is the vehicle’s frontal area; *α* is the road grade; and *v* is the velocity.

The bumper-to-bumper distance is shown between two platoon members with the decreased air drag coefficient as a percentage of the original [46]. For a platoon, the air drag coefficient is considered to be modified due to the different distance between the single movement status and platoon status. The form of the drag correction factor estimation model can be preliminary designed as [47].


$$\Gamma (y,A_{e},A_{f},A_{r})=1-ae^{by}\cdot \frac{A_{f}}{A_{e}}-ce^{dy}\cdot A_{r}$$ (5)

Where Γ represents the drag correction estimation value, which is the coefficient of the inter-vehicle distance between the front vehicle and the rear vehicle; *A*_*f*_ represents the cross-sectional area of the adjacent front vehicle; *A*_*r*_ represents the cross-sectional area of the adjacent rear vehicle; *y* is the inter-vehicle distance in the platoon; and *a*, *b*, *c*, *d* are the optimized parameters. The parameter identification of the model should be carried out separately according to the vehicles at different positions in the platoon.

The effect from both the inter-vehicle distances from the front vehicle and the rear vehicle is considered assuming that the reduction efficiency varies linearly as the inter-vehicle distances change [48].

The reduction efficiency of aerodynamic drag may vary depending on the position in the platoon. Therefore, the reduction efficiency function of the aerodynamic drag *E*_*i*_ of the *i*−*th* vehicle can be expressed as:

$$E_{i}=\{\begin{array}{l} \;\min\{\Gamma (y,A_{e},A_{r}),1\}\;i=1\\ \min\{\Gamma (y,A_{e},A_{f},A_{r}),1\}\;1<i<n\\ \;\min\{\Gamma (y,A_{e},A_{f}),1\}\;i=n \end{array}$$ (6)

As a result, the aerodynamic drag of the *i*−*th* vehicle can be calculated with Eq (6). The aerodynamic drag force *F*_*w*,*i*_ of the *i*−*th* vehicle in a platoon can be obtained as follows:

$$F_{w,i}=\frac{1}{2}E_{i}C_{D,i}A_{e,i}\rho v_{i}^{2}\;i=1,2,\cdots ,n$$ (7)

where *C*_*D*,*i*_ denotes the aerodynamic drag coefficient of the *i*−*th* vehicle; *A*_*e*,*i*_ denotes the cross-sectional area of the *i*−*th* vehicle in the platoon.

#### Powertrain system model

**Fig 1:**
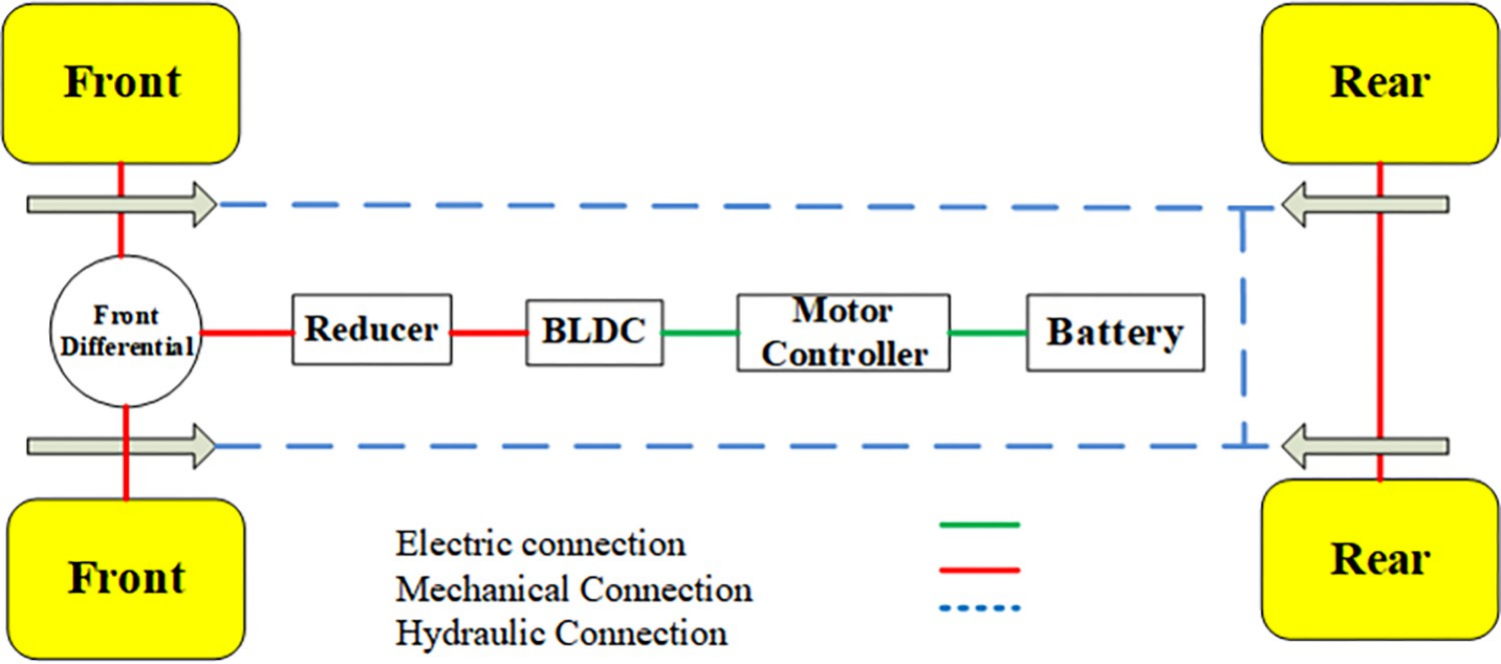
BEV powertrain.

**Fig 2:**
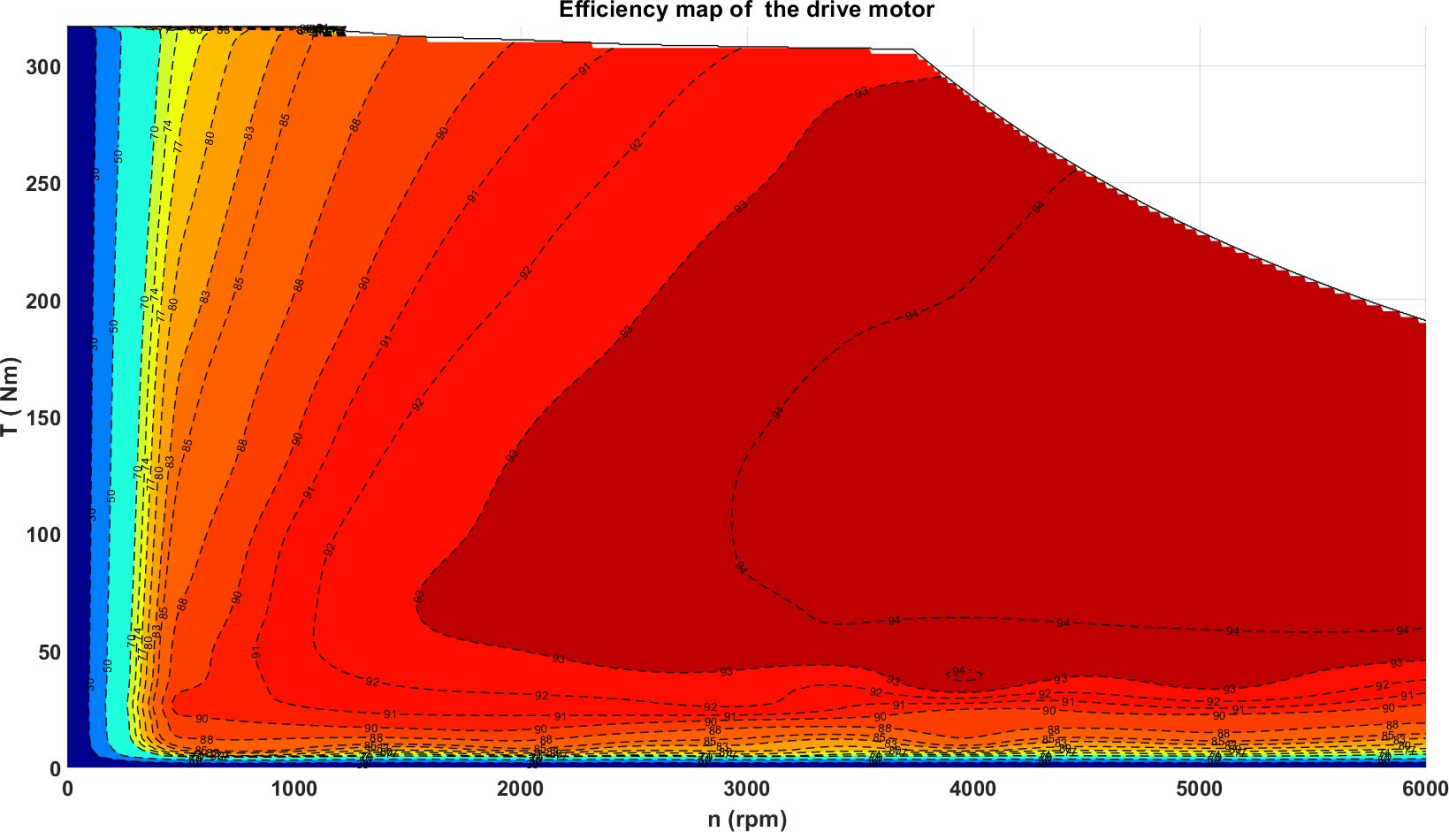
Efficiency map of drive motor.

The powertrain of a battery electric vehicle (BEV) for a brushless DC permanent magnet motor (BLDC) is shown in Fig 1, where the green lines and red lines represent the transfer of electrical and mechanical energy. The blue lines represent hydraulic connection. The power system consists of a power battery, drive motor, and control system. Electric motor converts electrical energy into mechanical energy to drive the wheels. Battery pack provides direct current (DC) electricity to power the vehicle’s systems and motor. The motor parameters and efficiency map are shown in Table 1 and Fig 2.

**Table 1:** The parameters of drive motor.

Motor parameters	value
Rated power, *Kw*	55
Peak power, *Kw*	120
Rated torque, *Nm*	180
Peak torque, *Nm*	350
Rated speed, *rpm*	3040
Peak speed, *rpm*	6000

The energy consumption of the drive motor of a BEV is considered in the Eco-CACC strategy as,

$$P(k|t)=\{\begin{array}{cc} \frac{T(k|t)v(k|t)}{r\eta_{t}\eta_{d}}, & T(k|t)\geq 0\\ \frac{T(k|t)v(k|t)\eta_{t}\eta_{b}}{r}, & T(k|t)<0 \end{array}$$ (8)

where *P*(*k*|*t*) denotes the motor power sequence and *k*∈[0,1,2,…,*N*_*p*_], which can be calculated according to the braking and driving conditions. *T*(*k*|*t*) denotes driving/braking torque sequence; *v*(*k*|*t*) denotes the speed sequence; *η*_*d*_ denotes the driving efficiency, and *η*_*b*_ denotes the braking efficiency of the motor.

For efficiency of a battery pack, a simplified static battery model is used in this article, and the gradient of SOC is formulated as follows [46],

$$\overset{•}{SOC}(t)=\frac{U_{oc}(SOC)-\sqrt{U_{oc}^{2}(SOC)-4P_{b}R_{b}(SOC)}}{2Q_{b}R_{b}(SOC)}$$ (9)

where *R*_*b*_ denotes the internal resistance, $U_{oc}^{2}(SOC)$ is the open-circuit voltage, *P*_*b*_ denotes output power of the battery, and *Q*_*b*_ is the nominal capacity of the battery.

#### Platoon model of nonlinear longitudinal dynamics

A nonlinear longitudinal dynamics model of a BEV in a platoon is established according to the longitudinal dynamic characteristics of the vehicle, as follows,

$$\left(\begin{array}{c} \overset{•}{p_{i}(t)}\\ \overset{•}{v_{i}(t)}\\ T_{i}(t) \end{array})=(\begin{array}{c} v_{i}(t)\\ \frac{\eta_{t}}{m_{i}r_{i}}T_{mi}(t)-\frac{1}{2m_{i}}\Omega v_{i}^{2}(t)-g\;\sin\;\alpha -gf\;\cos\;\alpha \\ -\tau_{i}\overset{•}{T_{i}(t)}+u_{i}(t-\theta_{i}) \end{array}\right)$$ (10)

where Ω = *C*_*D*,*i*_*A*_*e*,*i*_*ρ*. *p*_*i*_(*t*) denotes the position of the *i*−*th* vehicle at time *t*; *v*_*i*_(*t*) denotes the velocity of vehicle *i*−*th* at time *t*; *m*_*i*_ denotes the mass of the *i*−*th* vehicle; *r*_*i*_ is the tire radius of the *i*−*th* vehicle; *η*_*t*_ is the mechanical deficiency of the driving system; *τ*_*i*_ is the time constant of the actuator lag; *θ*_*i*_ is the input delay; and *T*_*i*_(*t*) is the driving/braking torque of the *i*−*th* vehicle. The state variables of a vehicle are the position, speed, and motor torque, and they are expressed as $x_{i}(t)=[p_{i}(t),v_{i}(t),T_{i}(t)]$. The control input *u*_*i*_(*t*) is expected to be the desired driving/braking torque. Here, *u*_*i*_(*t*) = *T*_*i*_(*t*).

Therefore, the dynamics of the following vehicles is $x_{i}(t+1)=A_{i}(x_{i}(t))+B_{i}u_{i}(t)=f(x_{i}(t),u_{i}(t))$, $y_{i}(t+1)=C_{i}(x_{i}(t))$. Where $y_{i}(t)≔[p_{i}(t),v_{i}(t)]$. $A_{i}={\left[p_{i}(t)+v_{i}(t)\Delta t,v_{i}(t)+\frac{\Delta t}{1.05}(\frac{\eta_{t}}{mR_{i}}T_{mi}(t)-gf-\frac{1}{2m}\Omega_{i}v_{i}^{2}(t)-g\;\sin\;\alpha (t)-gf\;\cos\;\alpha (t)),T_{i}(t)-\frac{1}{\tau_{I}}T_{i}(t)\Delta t\right]}^{T}$ where Δ*t* is the discrete time interval.


$$B_{i}={\left[0,0,\frac{\Delta t}{\tau_{i}}\right]}^{T},\;C_{i}=[\begin{array}{ccc} 1 & 0 & 0\\ 0 & 1 & 0 \end{array}].$$

### Merging and regrouped platoon model

#### Signal temporal logic

The cooperative control of platoon contains complex tasks. In order to solve traditional control objectives, general specifications are defined in temporal logic formulations that induce a sequence of control actions [25]. The signal temporal logic (STL) is beneficial as it is interpreted over continuous-time signals, allows for imposing tasks with strict deadlines, and introduces quantitative robust semantics [49, 50]. In this section, the STL fragment is introduced. The STL is based on predicates *μ* that are obtained after evaluation of a continuously differentiable predicate function *h*:ℝ^*m*^→ℝ as $\mu ≔\left\{ \begin{array}{c} ⊤,\;h(x)\geq 0\\ ⊥,\;h(x)<0 \end{array}\right.$ for *x*∈ℝ^*m*^. Notations ⊤ and ⊥ donate the Boolean ‘true’ and ‘false’, respectively. ℝ are the real numbers, while ℝ^*m*^ is the *m* dimensional real vector space. The non-negative real numbers ℝ_≥0_.

**Definition 1** (STL Syntax): The STL Syntax [50–52] is recursively defined by:

$$\phi :≔⊤|\mu |\neg \phi |\phi_{1}\wedge \phi_{2}|\phi_{1}U_{\left[\tau_{1},\tau_{2}\right]}\phi_{2}$$

where *ϕ*_1_,*ϕ*_2_ are the STL formulas. The satisfaction relation (*x*,*t*)| = *ϕ* denotes if the signal *x*:ℝ_≥0_→ℝ^*n*^, satisfies *ϕ* at time *t*. Notations ¬ and ∧ are the Boolean ‘negation’ and ‘conjunction’, respectively. $U_{\left[\tau_{1},\tau_{2}\right]}$ denotes until operator, it specifies that a condition holds true until another condition becomes true over the interval [*τ*_1_, *τ*_2_], *τ*_1_, *τ*_2_∈ℝ_≥0_ with 0≤*τ*_1_≤*τ*_2_<∞.

#### Merging communication structure

The PLF topology and a connected and undirected graph are applied to model the merging control.

The regroup platoon consists of an original platoon and a sub-platoon which contains two vehicles. This structure ensures a consistent connection among the ego vehicle, the predecessor vehicle, and the leading vehicle. This connection is likely crucial for maintaining coordination within the platoon.

A connected and directed graph G is defined as G≔(V,C), V≕{1,2,⋯,n} indicates the set consisting of leader, ego and its predecessor, ego and its follower for the regrouped platoon size. C≔{(i,j)|i,j∈V,i≠j} donates the edge set. C∈V×V represents communication links during merging task. The sub-platoon set is denoted by $N_{i}=\{j\in V|(i,j)\in C,i\neq j\}$. It is supposed that vertices sets *V*_*f*_≔{1,2,⋯,*n*_*f*_} are predecessor-followers. And set *V*_*l*_≕{*n*_*f*_+1,*n*_*f*_+*n*_*l*_} are leaders for an original platoon and a sub-platoon, *n* = *n*_*f*_+*n*_*l*_.

The merging task structure can be presented according to the Laplacian matrix as,

$$L_{indvs}=[\begin{array}{cc} \Lambda_{pf} & \Lambda_{lf}\\ \Lambda_{fl} & \Lambda_{ll} \end{array}]$$ (11)

where **Λ**_***pf***_ represents the Laplacian matrix of predecessor-followers communication; **Λ**_***lf***_ represents the communications from the leader to its followers; and **Λ**_***plf***_ represents the communications from the predecessors to leader. It is assumed that the communication between the leader and its follower can be directed, and **Λ**_***fl***_≠**Λ**_***lf***_^***T***^, but vice versa **Λ**_***pf***_ = ***0***. **Λ**_***ll***_ demonstrates the communications both the leaders of original platoon and sub-platoon before pre-merging task. The communication both the leaders of platoons in a regrouped platoon is assumed to be directed. However, the original platoon and sub-platoon are independent to their local tasks before pre-merging task. It is assumed that there is no coordination, **Λ**_***ll***_ = ***0***.

According to Eq (10), *p*_*i*_(*t*)∈ℝ, *v*_*i*_(*t*)∈ℝ, *u*_*i*_(*t*)∈ℝ of vehicle i∈V. $N_{i}$ denotes the set of neighbors of the vehicle *i*, and $\left|N_{i}\right|$ denotes the cardinality of the set $N_{j}$. The second-order dynamics of vehicle *i* for the predecessor-followers is expressed as follows,

$$[\begin{array}{c} \overset{•}{p_{i}}(t)\\ v_{i}(t) \end{array}]=[\begin{array}{c} v_{i}(t)\\ f_{i}^{d}(p_{i}(t),v_{i}(t)) \end{array}],\;i\in \{1,2,\cdots ,n_{f}\}$$ (12)

The second-order dynamics of vehicle *i* for leaders is expressed as,

$$[\begin{array}{c} \overset{•}{p_{i}}(t)\\ v_{i}(t) \end{array}]=[\begin{array}{c} v_{i}(t)\\ f_{i}^{d}(p_{i}(t),v_{i}(t)) \end{array}]+[\begin{array}{c} 0\\ g_{i}^{d}(v_{i}(t))u_{i}(t) \end{array}],\;i\in \{n_{f}+1,n_{f}+n_{l}\}$$ (13)

where $f_{i}^{d}(p_{i}(t),v_{i}(t)):{\mathbb{R}}^{2+2|N_{i}|}\to \mathbb{R},g_{i}^{d}(v_{i}(t)):\mathbb{R}\to \mathbb{R}$ are respected to be Lipschitz continuous. Definition the second order dynamics are guaranteed [44]. Based on Eqs (12) and (13), the stacked dynamics for the set of vehicles in i∈V, as

$$\overset{•}{x^{d}}=f^{d}(x^{d})+g^{d}(x^{d})u$$ (14)

Where $x^{d}≔{\left[x^{d}\right]}_{i\in V}=[\begin{array}{c} p_{i}(t)\\ v_{i}(t) \end{array}]\subseteq {\mathbb{R}}^{2m}$. $f^{d}(\cdot )≔{\left[f_{i}^{d}(\cdot )\right]}_{i\in V}\in {\mathbb{R}}^{2m}$, $g^{d}(\cdot )≔[\begin{array}{c} 0_{n_{f}\times n_{l}}^{Τ}\\ g_{n_{l}}(\cdot )I_{n_{l}} \end{array}]$, $g_{n_{l}}(\cdot )≔{\left[g_{i}(\cdot )\right]}_{i\in (n_{f}+1,\cdots ,n_{f}+2)}$. The input matrices $g_{n_{l}}(\cdot )$ is not full row rank. The control input signal is $u≔{\left[u_{i}(t)\right]}_{i\in (n_{f}+1,n_{f}+2)}\in {\mathbb{R}}^{n_{l}}$.

### Performance of the Eco-CACC merging strategy

In each prediction time domain of the DNMPC-STL, the leader and the predecessor-followers are regarded as multi-objective tasks that can be used to establish multi-objective functions and constraints for the regrouped platoon. The stability, energy consumption and tracking performance are evaluated based on the safety of the merging platoon.

Considering that the strategy of merging target is involved in the process of regrouping the platoon, assumptions on the basis of the Eco-CACC system are made.

Assumptions:

1. All vehicles get the leader’s information either directly or indirectly stability in platooning;
2. During the process of merging the platoon, the platoon meets the conditions of speed changes at a stable range;
3. The process of merging the platoon ignores external environmental interference factors.

#### Platoon merging controller of leaders

The energy consumption objective is expressed as,

$$J_{l}^{e}(k|t)={\left\|W_{1}^{i}P_{1}^{i}(k|t)\Delta t\right\|}_{2}+{\left\|W_{1}^{j}P_{1}^{j}(k|t)\Delta t\right\|}_{2}$$ (15)

where $J_{l}^{e}(k|t)$ represents the energy consumption of leaders and Δ*t* represents the time steps of leaders. $P_{1}^{i}(k|t)$ represents the energy consumption of the original platoon leader (OPL) during the prediction time steps *N*_*p*_. $P_{1}^{j}(k|t)$ represents the energy consumption of the sub-platoon leader (SPL). $W_{1}^{i}$ and $W_{1}^{j}$ represent the weight coefficients of the energy consumption of OPL and SPL, respectively.

For a leader, the speed fluctuation should remain as low as possible to ensure motor torque stability. The comfort objective function $J_{l}^{c}(s|t)$ is expressed as follows:

$$J_{l}^{c}(k|t)={\|R_{1}^{i}(T_{1}^{i}(k|t)-T_{1}^{j}(v_{1}^{i}(k|t)))\|}_{2}+{\|R_{1}^{j}(T_{1}^{j}(k|t)-T_{1}^{j}(v_{1}^{j}(k|t)))\|}_{2}$$ (16)

where $J_{l}^{s}(k|t)$ represents the merging comfort cost of the leaders. $T_{1}^{i}\;(v_{1}^{i}(k|t))$ and $T_{1}^{j}(v_{1}^{j}(k|t))$ represent the torques at stable OPL and SPL speeds, respectively. $R_{1}^{i}$ and $R_{1}^{j}$ are the weight coefficients of the driving stability of the leaders of the original platoon and sub-platoon, respectively. These parameters can be expressed as follows:

$$T(v(N_{p}|t)=\frac{r\eta_{d}}{i_{g}}(\frac{1}{2}\Omega {\left(v\right)}^{2}(N|t)-mgf-1.05m\frac{dv(N_{p}|t)}{dt})$$ (17)

#### Platoon merging controller of the predecessor-followers

The energy consumption of the predecessor-followers for the merging task as follows:

$$J_{i}^{e}(k|t)={\left\|W_{i}P_{i}(k|t))\Delta t\right\|}_{2}$$ (18)

where $J_{i}^{e}(k|t)$ represents the energy consumption of the predecessor-followers, *W*_*i*_ represents the weight coefficient of the energy consumption, and *p*_*i*_(*k*|*t*) represents motor power of the predecessor-followers.

The driving stability cost function $J_{i}^{c}(s|t)$ is expressed as follows:

$$J_{i}^{c}(k|t)={\|R_{i,1}(T_{1}\;(k|t)-T_{i}(v_{i}(k|t)))\|}_{2}+{\left\|R_{i,i-1}(T_{i,i-1}(k|t)-T_{i}(v_{i}(k|t)))\right\|}_{2}$$ (19)

where $J_{i}^{c}(k|t)$ represents the driving stability cost of vehicle *i*; *T*_1_(*k*|*t*) represents the desired torque of the original leader, which controls the leader vehicle; and *T*_*i*,*i*−1_(*k*|*t*) represents the desired torque of Vehicle *i*, which controls vehicle (*i*−1). *T*_*i*_(*v*_*i*_(*k*|*t*)) represents the torque of vehicle *i* at a stable speed, which is a constant term. *R*_*i*,1_ represents the weight coefficient matrices of the stability according to the original leader state. *R*_*i*,*i*−1_ represents the weight coefficient matrices according to the predecessor (*i*−1) state.

Ensuring precise vehicle tracking performance in predecessor-following contributes significantly to the success of platooning technology and its adoption in real-world scenarios. Platooning systems are based on robust control strategies and technologies to enhance the safety and stability of platoons on the road.

Therefore, $y_{i}(k)={\left[p_{i}(k),v_{i}(k),T_{i}(k)\right]}^{⊤}$ denotes the real driving-state sequence of vehicle *i*.The desired state that the ego’s following vehicles aim $y_{i,1}^{a}(k)$ follow and the expected control inputs are depicted. The desired state sequence of the ego can be predicted according to the leader’s state as $y_{i,1}^{a}(k)={\left[p_{i,1}^{a}(k),v_{i,1}^{a}(k),T_{i,1}^{a}(k)\right]}^{⊤}={\left[p_{1}^{a}-(i-1)d_{i,i-1},v_{1}^{a},T_{1}^{a}\right]}^{⊤}$. The desired state sequence $y_{i,i-1}^{a}(k)$ of the ego can be predicted according to the predecessor’s state as $y_{i,i-1}^{a}(k)={\left[p_{i-1}^{a}(k),v_{i-1}^{a}(k),T_{i-1}^{a}(k)\right]}^{⊤}={\left[p_{i-1}^{a}-d_{i,i-1},v_{i-1}^{a},T_{i-1}^{a}\right]}^{⊤}$, *d*_*i*,*i*−1_ is the desired safety distance between the ego and its follower.

The tracking objective function of vehicle *i* is expressed as follows:

$$J_{i}^{t}(k|t)={\|Q_{i,1}(y_{i}(k|t)-y_{i,1}^{a}(k|t))\|}_{2}+{\|Q_{i,i-1}(y_{i}(k|t)-y_{i,i-1}^{a}(k|t))\|}_{2}$$ (20)

where $J_{i}^{t}(s|t)$ represents the tracking cost of vehicle *i*, *Q*_*i*,1_ represents the weight coefficient according to the leader state, and *Q*_*i*,*i*−1_ represents the weight coefficient according to the predecessor (*i*−1). The desired state $y_{i,1}^{a}(k|t)$ of the ego denotes the information delivery state according to the original leader’s state. The desired state $y_{i,i-1}^{a}(k)$ of the ego denotes the information delivery state according to the predecessor’s state.

The desired state that the ego’s following vehicles aim to follow and the expected control inputs are expressed:

$$J_{i}^{m}(k|t)={\|F_{i,1}(y_{j}(k|t)-y_{i,1}^{a}(k|t)-d_{j,1})\|}_{2}+{\|F_{i,i-1}(y_{j}(k|t)-y_{i,i-1}^{a}(k|t)-d_{j,i})\|}_{2}$$ (21)

where $J_{i}^{m}(k|t)$ represents the merging error of vehicle *i*. $d_{j,i}={\left[(j-i)d_{i,i-1},0\right]}^{⊤}$ is the deviation of the merging distance between vehicle *j* in sub-platoon and vehicle *i* in original platoon.

#### Objective function and constraints of the Eco-CACC merging strategy

Taking all objective functions into consideration, the DNMPC-STL controller is expressed as,

$$\min\;J=\sum_{i=0}^{N_{p}-1}J_{l}^{e}(s|t)+J_{l}^{c}(k|t)+J_{i}^{e}(k|t)+J_{i}^{c}(k|t)+J_{i}^{t}(k|t)+J_{i}^{m}(k|t)$$ (22)

$$s.t.\;x_{i}(k+1|t)=A_{i}x_{i}(k|t)+B_{i}u_{i}(k|t)$$

$$y_{i}(k+1|t)=C_{i}x_{i}(k|t)$$

$$p_{i}(N_{p}|t)=p_{1}(N_{p}|t)-id_{i,i-1}$$

$$v_{\min}\leq v(k|t)\leq v_{\max}$$

$$T_{\min}\leq T(k|t)\leq T_{\max}$$

$$y_{i}(N_{p}|t)=\frac{1}{\left|\Theta_{i}\right|}\sum_{j\in C}\left(y_{j}(N_{p}|t)-d_{j,i}\right)$$

$$T_{i}(N_{p}|t)=T_{i}(v_{i}(N_{p}|t))$$

Where, control optimized sequence is $U_{i}={\left[u_{i}(0|t),u_{i-1}(0|t),u_{i}(0|t),\cdots ,u_{i}(N_{p}-1|t)\right]}^{⊤}$. The Θ_*i*_ donates the set of vehicles that can receive status information. |Θ| is the cardinality of set Θ_*i*_; The terminal constraint Eq (22) represent respectively: dynamics constrains in the prediction time domain, control output, speed constrains, torque constrains, deviation constrains of the merging distance, the terminal state in the predictive horizon. The terminal constraint is to keep the same output that sub-platoon’s vehicle merge into original platoon at the end of horizon. The terminal constraint is to keep that the platoon meets the conditions of speed changes at a stable range during the merging process at the end of predictive horizon.

#### The stability analysis based on Lyapunov theory

Analogous to Lyapunov stability [53], Lyapunov string stability (LSS) and asymptotic Lyapunov string stability (ALSS) are proposed.

**Definition 2.** The equilibrium point *x*_*e*_ of a platoon system is said to be LSS if for each *ϵ*>0, ∃*δ* = *δ*(*ϵ*)>0,

$${\left\|x(0)\right\|}_{2}<\delta \Rightarrow {\left\|x(t)\right\|}_{2}<\epsilon \forall t\geq 0,\forall m\in \mathbb{N}$$ (23)

**Definition 3.** The equilibrium point *x*_*e*_ of a platoon system is said to be ALSS if it is LSS and *x*(*t*)→0 asymptotically.

**Assumption 1** [28] The directed graph G of the platoon topology contains a spanning tree rooted at the lead vehicle.

**Lemma 1** [28, Theorem]: If Assumption 1 is satisfied, the Eq (22) guarantee convergence of the output to the desired output in at most N steps, that is

$$\underset{t\to \infty }{\lim}|y_{i}(N_{p}|t)-y_{i,1}^{a}(N_{P}|t)=0,t\geq N$$ (24)

$$\underset{t\to \infty }{\lim}|y_{i}(N_{p}|t)-y_{i,i-1}^{a}(N_{P}|t)=0,t\geq N$$ (25)

**Lemma 2** ([28, Theorems 3 and 4]): The objective function in Eq (22) is the sum of non-increasing Lyapunov functions; hence, this system is asymptotically stable.

The optimal cost function value of vehicle *i* at time *t* can be obtained as,

$$\begin{array}{l} J_{i}^{*}(t)=(y_{i}^{*}(:|t),u_{i}^{*}(:|t),y_{i}^{a}(:|t),y_{j}^{*}(:|t),y_{1}^{a}(:|t)\\ \;=\sum_{k=0}^{N_{p}-1}\{J_{l}^{e}(k|t)+J_{l}^{c}(k|t)+J_{i}^{e}(k|t)+J_{i}^{c}(k|t)+J_{i}^{t}(k|t)+J_{i}^{m}(k|t)\} \end{array}$$ (26)

At the moment *t*+1, the cost function to be optimized is given as follows,

$$J_{i}^{*}(t+1)\leq l_{i}(y_{i}^{*}(:|t+1),u_{i}^{*}(:|t+1),y_{i}^{a}(:|t+1),y_{j}^{a}(:|t+1),y_{1}^{a}(:|t+1))$$ (27)

Then,

$$\begin{array}{l} J_{i}^{*}(t+1)-J_{i}^{*}(t)\leq -l_{i}(x_{i}^{*}(0|t),u_{i}^{*}(0|t),x_{i}^{a}(0|t),x_{j}^{a}(0|t)+x_{1}^{a}(0|t))\\ +[l_{i}(x_{i}^{*}(k|t),u_{i}^{*}(k|t),x_{i}^{*}(k|t),x_{j}^{*}(k|t),x_{1}^{*}(k|t))\\ -l_{i}(x_{i}^{*}(k|t),u_{i}^{*}(k|t),x_{i}^{a}(k|t),x_{j}^{a}(k|t),x_{1}^{a}(k|t))] \end{array}$$ (28)

The last item in Eq (28), the triangle inequality using the norm,

$$\begin{array}{l} l_{i}(x_{i}^{*}(k|t),u_{i}^{*}(k|t),x_{i}^{*}(k|t),x_{j}^{*}(k|t),x_{1}^{*}(k|t))-l_{i}(x_{i}^{*}(k|t),u_{i}^{*}(k|t),x_{i}^{a}(k|t),x_{j}^{a}(k|t),x_{1}^{a}(k|t))\\ =\sum_{i=1}^{N_{p}}\left(\begin{array}{l} \|Q_{i,1}(y_{j}^{*}(k|t)-y_{i,1}^{*}(k|t)){\|}_{2}+\|Q_{i,i-1}(y_{j}^{*}(k|t)-y_{i-1,i}^{a}(k|t)){\|}_{2}\\ -\|Q_{i,1}(y_{j}^{*}(k|t)-y_{i-1,i}^{*}(k|t)){\|}_{2}-\|Q_{i,i-1}(y_{j}^{*}(k|t)-y_{i-1,i}^{a}(k|t)){\|}_{2} \end{array}\right)\\ +\sum_{i=1}^{N_{p}}\left(\begin{array}{l} \|F_{i,1}(y_{j}^{*}(k|t)-y_{i,1}^{*}(k|t)){\|}_{2}+\|F_{i,i-1}(y_{j}^{*}(k|t)-y_{i-1,i}^{*}(k|t)){\|}_{2}\\ -\|F_{i,1}(y_{j}^{*}(k|t)-y_{i,1}^{a}(k|t)-d_{i,1}){\|}_{2}-\|F_{i,i-1}(y_{j}^{*}(k|t)-y_{i,i-1}^{a}(k|t)-d_{i-1,i}){\|}_{2} \end{array}\right)\\ \leq \sum_{i=1}^{N_{p}}\left({\left\|Q_{i,1}(y_{i-1,i}^{*}(k|t)-y_{i,1}^{*}(k|t))\right\|}_{2}+{\left\|F_{i,1}(y_{i,1}^{a}(k|t)-y_{j}^{*}(k|t))\right\|}_{2}+{\left\|F_{i,i-1}(y_{i-1,i}^{a}(k|t)-y_{j}^{*}(k|t))\right\|}_{2}\right) \end{array}$$ (29)

$J_{\Sigma }^{*}(t)$ donates the sum of the optimal values of all vehicles at moment *t*, then

$$J_{\Sigma }^{*}(t+1)-J_{\Sigma }^{*}(t)\leq -\sum_{i=1}^{N}l_{i}(y_{i}^{*}(1|t),u_{i}^{*}(0|t),y_{i}^{a}(1|t),y_{-i}^{a}(1|t))+\sum_{k=1}^{N_{p}-1}\varepsilon_{\Sigma }(k)$$ (30)

$$\sum_{k=1}^{N_{p}-1}\varepsilon_{\Sigma }(k)=\sum_{j\in \Theta }(\sum_{i=1}^{N}\left(\begin{array}{l} {\|Q_{i,1}(y_{i-1,i}^{*}(k|t)-y_{i,1}^{*}(k|t))\|}_{2}+{\left\|F_{i,1}(y_{i,1}^{a}(k|t)-y_{j}^{*}(k|t))\right\|}_{2}\\ +{\left\|F_{i,i-1}(y_{i-1,i}^{a}(k|t)-y_{j}^{*}(k|t))\right\|}_{2} \end{array}\right)$$ (31)

Where, Θ_*i*_ donates the set of vehicles that can receive status information.

According to the LSS, when $J_{\Sigma }^{*}(t+1)<J_{\Sigma }^{*}(t)$, the asymptotic stability of the platoon can be achieved, the $\sum_{k=1}^{N_{p}-1}\varepsilon_{\Sigma }(k)<0$ needs to be satisfied by artificially setting and values, so that the asymptotic stability of *Q*_*i*,1_, *F*_*i*,1_ and *F*_*i*,*i*−1_.

## Analysis

In most existing studies, merging positions are located at the head, middle, and tail of a platoon. The choice of merging position can be based on application needs. The platoon leader may permit vehicles to merge at the tail to reduce information exchange and impact on the existing vehicles in the platoon as much as possible. To enhance operational flexibility while keeping the platoon leader unchanged, merging in the middle can be allowed. In this section, numerical experiments are performed to evaluate the performances of the two merging positions. Compared with the two merging positions, the Eco-CACC merging strategy based on the DNMPC-STL approach is proposed for evaluating stability, tracking performance and SOC of battery.

**Fig 3:**
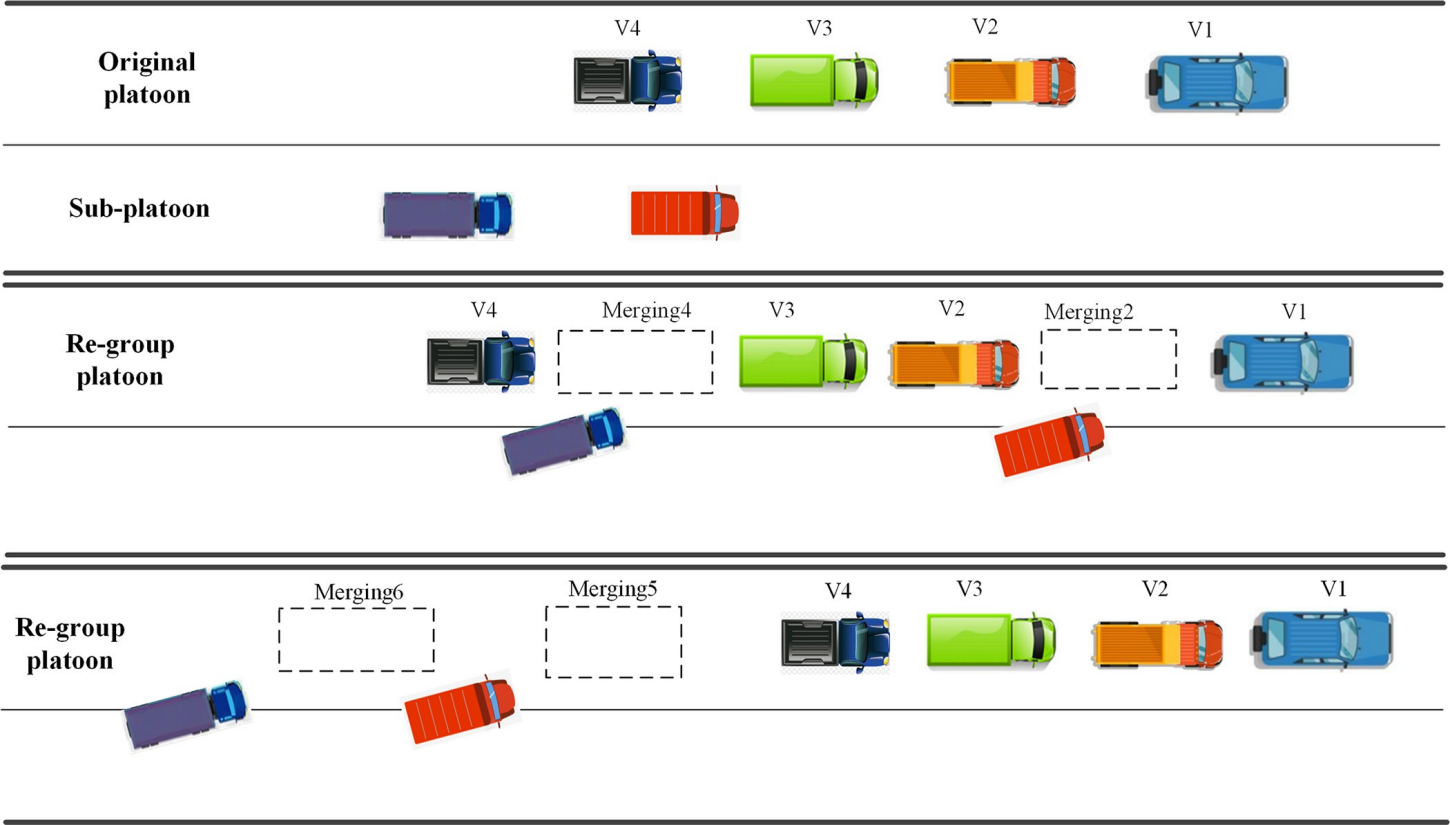
Merging task for style 1 and style 2. (a) Pre-merging task. (b) Re-group platoon of merging task for style 1. (c) Re-group platoon of merging task for style 2.

We have chosen to evaluate merging performance in the middle (style) and at the tail (style) of the original platoon. Style 1 is described in Fig 3(A) and 3(B). In this example, an original platoon consists of four vehicles, the sequence is {Vehicle 1, Vehicle 2, Vehicle 3, Vehicle 4}. The sub-platoon consists of two vehicles. In-vehicle distances are available for merging task in the original platoon. Then, the sub-platoon merges ahead of Vehicle 2 and Vehicle 4, respectively, which are named the position sequence {Merging 2, Merging 4}. Style 2 is described as Fig 3(A) and 3(C). The sub-platoon is available for following at the end position of the original platoon, where are named as position sequence {Merging 5, Merging 6}.

In the style 1, according to Eq (11), the Laplacian matrix of the original platoon and sub-platoon network can be specified by

$$L_{indvs}=[\begin{array}{cccccc} 3 & 0 & -1 & -1 & 0 & -1\\ 0 & 1 & 0 & 0 & -1 & 0\\ 0 & 0 & 2 & -1 & 0 & -1\\ 0 & 0 & 0 & 1 & 0 & -1\\ 0 & -1 & 0 & 0 & 1 & 0\\ 0 & 0 & 0 & 0 & 0 & 1 \end{array}]$$

According to Eq (14), where $f_{i}^{d}(p_{i}(t),v_{i}(t))≔[\begin{array}{cc} 0_{6\times 6} & I_{6\times 6}\\ -L_{indivs} & -L_{indivs} \end{array}][\begin{array}{c} p_{i}(t)\\ v_{i}(t) \end{array}]$, and $g_{i}^{d}(v_{i}(t))≔[\begin{array}{c} 0_{12}^{Τ}\\ 1_{2}^{Τ} \end{array}]$ The merging task of the style 1 could specified as the STL semantics as,

$$\begin{array}{l} \phi_{PFL-merge}=(\|p_{1}(t)-p_{merg\mathrm{ing}2}(t)\leq \varsigma_{m}\|)\wedge (\|p_{1}(t)-p_{merging2}(t)\geq \varsigma_{s}\|)U_{\left[\tau_{1},\tau_{2}\right]}\\ \;(\|p_{3}(t)-p_{merging4}(t)\leq \varsigma_{m}\|)\wedge (\|p_{3}(t)-p_{merging4}(t)\geq \varsigma_{s}\|) \end{array}$$

Where *ς*_*m*_ is the distance threshold for pre-merging task; *ς*_*s*_ represents a safety distance of collision avoidance under merging. The time internal is [*τ*_1_, *τ*_2_].

The Laplacian matrix of the regrouped platoon under PLF topology can be expressed by

$$L_{PLF-merge}=[\begin{array}{cccccc} 5 & -1 & -1 & -1 & -1 & -1\\ 0 & 2 & -1 & 0 & -1 & 0\\ 0 & 0 & 1 & -1 & 0 & 0\\ 0 & 0 & 0 & 2 & -1 & -1\\ 0 & 0 & 0 & 0 & 1 & -1\\ 0 & 0 & 0 & 0 & 0 & 1 \end{array}]$$

In the style 2, the Laplacian matrix of the original platoon and sub-platoon network can be specified by

$$L_{indvs}^{*}=[\begin{array}{cccccc} 3 & -1 & -1 & -1 & 0 & 0\\ 0 & 2 & -1 & -1 & 0 & 0\\ 0 & 0 & 1 & -1 & 0 & 0\\ 0 & 0 & 0 & 1 & 0 & 0\\ 0 & 0 & 0 & 0 & 1 & -1\\ 0 & 0 & 0 & 0 & 0 & 1 \end{array}]$$

Where $f_{i}^{d}(p_{i}(t),v_{i}(t))≔[\begin{array}{cc} 0_{6\times 6} & I_{6\times 6}\\ -L_{indivs}^{*} & -L_{indivs}^{*} \end{array}][\begin{array}{c} p_{i}(t)\\ v_{i}(t) \end{array}]$, and $g_{i}^{d}(v_{i}(t))≔[\begin{array}{c} 0_{12}^{Τ}\\ 1_{2}^{Τ} \end{array}]$ Regarding the merging task, the style 2 could specified as the STL semantics.


$$\phi_{merge}^{*}=(\|p_{Merging5}(t)-p_{4}(t)\|\geq \varsigma_{m})U_{\left[\tau_{1},\tau_{2}\right]}(\|p_{Merging6}(t)-p_{Merging5}(t)\|\geq \varsigma_{m})$$

According to Eq (14), the Laplacian matrix of the regrouped platoon of style 2 under PLF topology can be expressed by

$$L_{PLF-merge}^{*}=[\begin{array}{cccccc} 5 & -1 & -1 & -1 & -1 & -1\\ -1 & 1 & 1 & 0 & 0 & 0\\ -1 & -1 & 1 & 1 & 0 & 0\\ -1 & 0 & -1 & 1 & 1 & 0\\ -1 & 0 & 0 & -1 & 1 & 1\\ -1 & 0 & 0 & 0 & -1 & 2 \end{array}]$$

In this simulation environment, the road grade is set to *α* = 0. The maximum velocity is 120km/h. In Eq (5), four drag correction factors are optimized, *a* = 0.4, *b* = 0.022, *c* = 0.11, *d* = 0.28. The coefficient of rolling resistance is set to *f* = 0.015. The STL time internals are *τ*_1_ = 4 *τ*_2_ = 12 *τ*_3_ = 4 *τ*_4_ = 12. For the merging task, *ς*_*m*_ = 20, *ς*_*s*_ = 15, *ς*_*l*_ = 3. The initial SOC of battery is 0.65. The battery capacities of all members are 13.1kwh, voltages are 237V.

## Results

**Fig 4:**
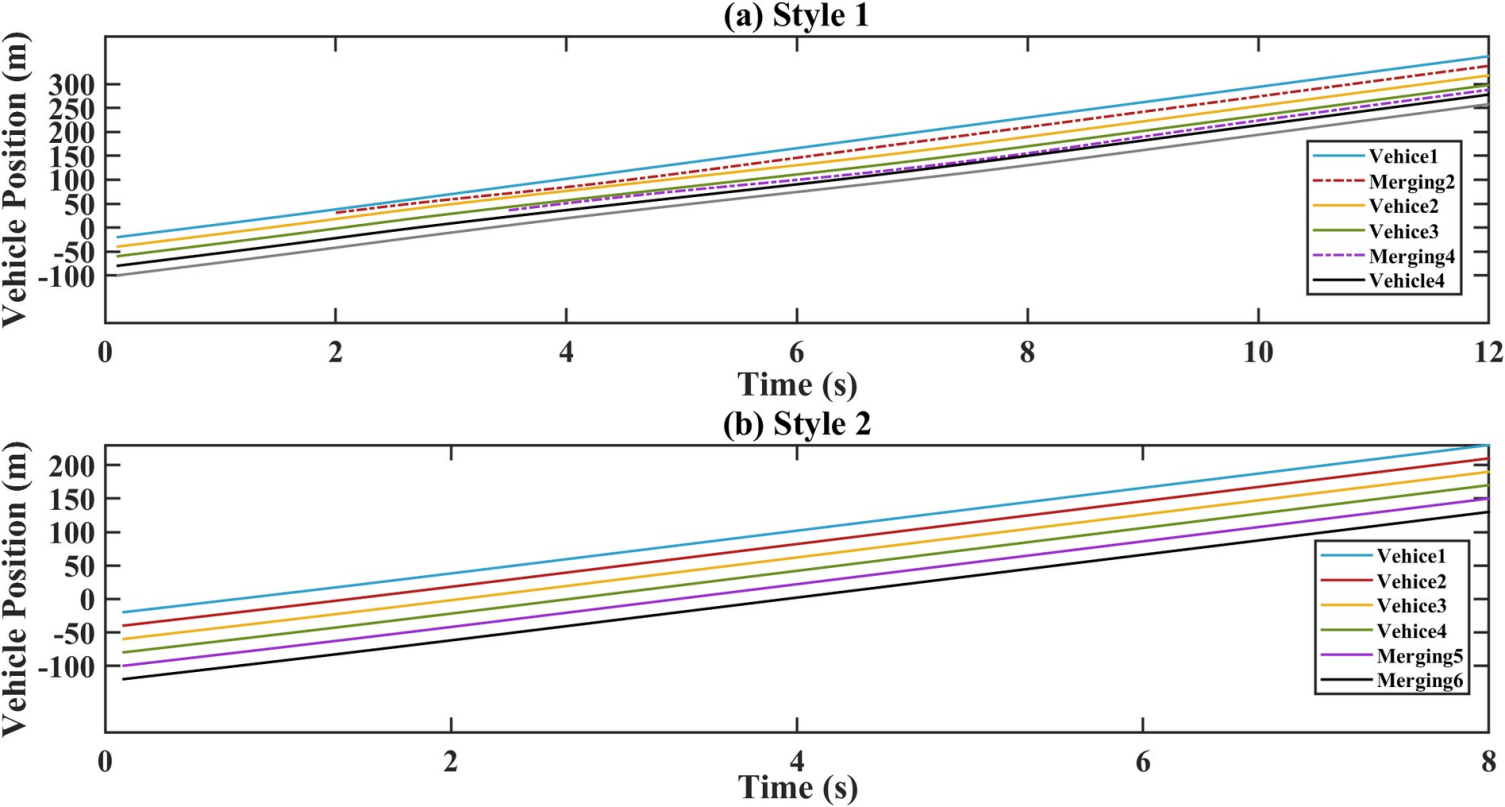
Spatiotemporal trajectory diagram. (a) Style 1; (b) Style 2.

According to the experimental design in Section 3, the proposed platoon merging strategy was simulated for two merging positions. The parameters are shown in Tables 2 and 3. The spatiotemporal trajectories for both style 1 and style 2 are shown in Fig 4(A) and 4(B). Both style 1 and style 2, no collisions occurred in the new platoon. These trajectories illustrate the relationship between the merging positions and time.

**Table 2:** The parameters of the platoon.

Value Symbol	Original platoon	Sub-platoon
*m*_*i*_, kg	3495, 2400, 3575, 3295	2510, 2350
*C*_*Di*_	1.69, 1.12, 1.56, 1.15	1.09, 1.61
*r*_*i*_, m	0.31, 0.39, 0.37, 0.38	0.38, 0.36
*A*_*i*_, m^2^	4.45, 4.25, 5.9, 5.75	4.45, 4.20
*τ*_*i*_,*s*	0.24, 0.23, 0.22, 0.26	0.22, 0.24
*θ*_*i*_,*s*	0.23,0.27,0.24,0.20	0.20, 0.23
*η*_*t*_	095, 0.96, 0.93, 0.94	0.93, 0.94

**Table 3:** Weights of the cost functions.

Weights	PFL weights	
*W*	*W*_1_ = 10*I*_2_	*W*_*i*_ = 10*I*_2_
*R*	*R*_1_ = 0	*R*_*i*,1_ = *R*_*i*,*i*−1_ = 5*I*_2_
*Q*	*Q*_1_ = 0	*Q*_*i*,1_ = *Q*_*i*,*i*−1_ = 2*I*_2_
*F*	*F*_1_ = 0	*F*_*i*,1_ = *F*_*i*,*i*−1_ = 8*I*_2_

**Fig 5:**
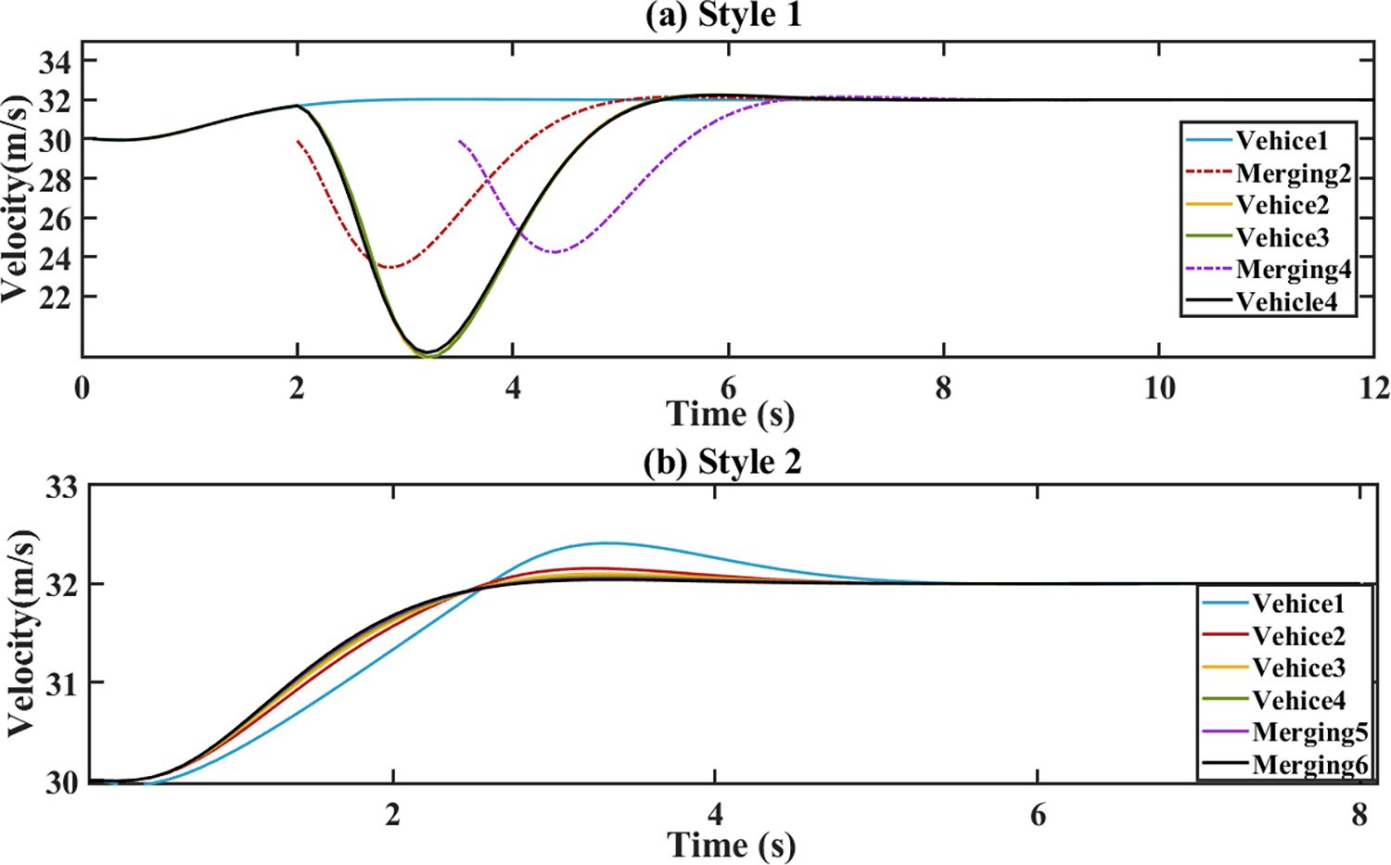
Velocity. (a) Style 1. (b) Style 2.

The simulation results illustrate the velocity of vehicles as shown in Fig 5(A) and 5(B). In the ahead of Vehicle 2 and Vehicle 4, positions of‘Merging 2’ and‘Merging 4’ are respectively occupied by sub-platoon at the desired gap, members of new platoon maintain PFL topology. The original platoon increases the ahead gaps of the predicted merging positions by reducing the velocity. The proposed cooperative merging method ensure that the speed fluctuations among the following vehicles are within a small range for style 1 during 2-6s. This approach is beneficial for maintaining smooth and coordinated motion within the platoon.

**Fig 6:**
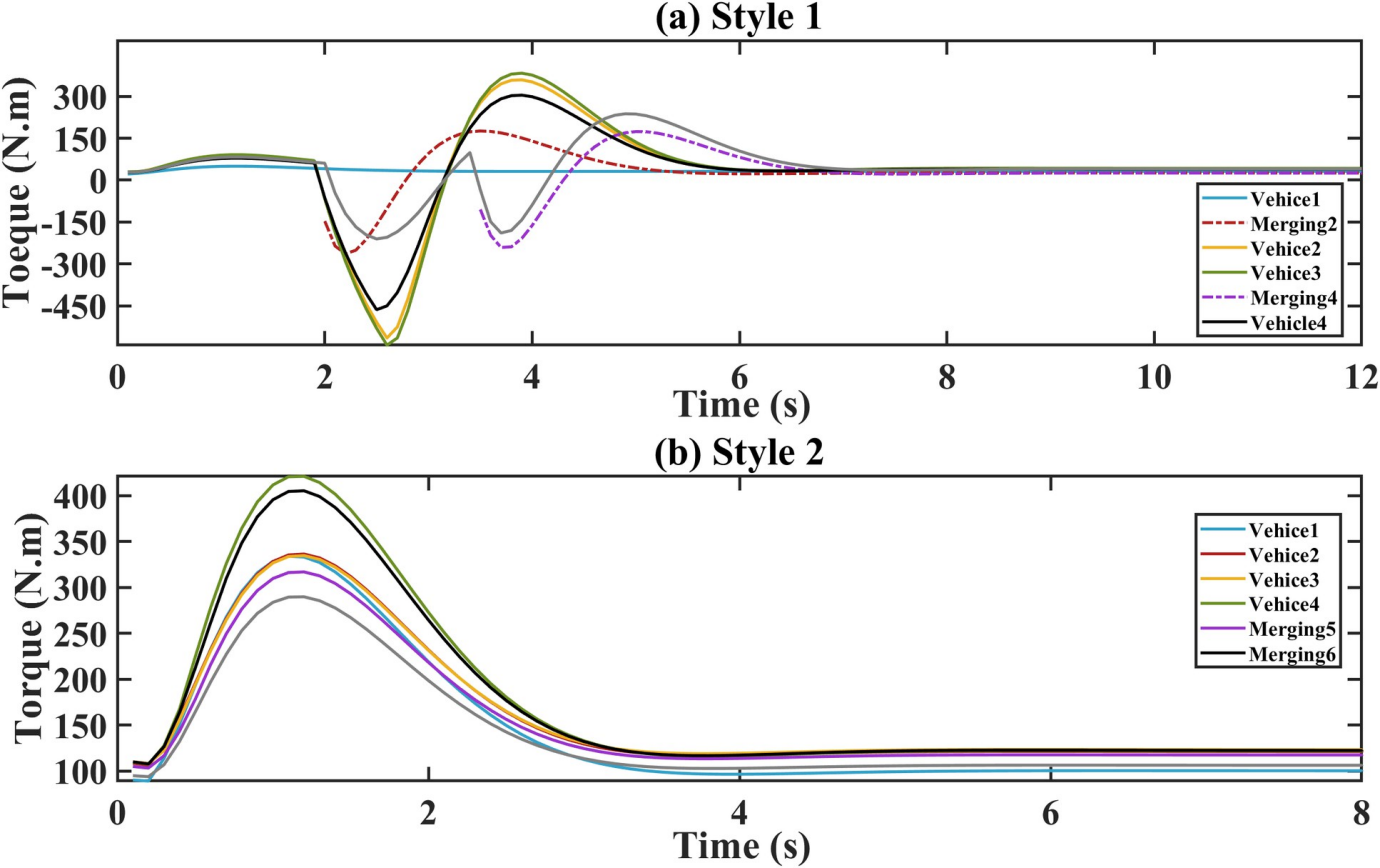
Torque. (a) Style 1; (b) Style 2.

**Fig 7:**
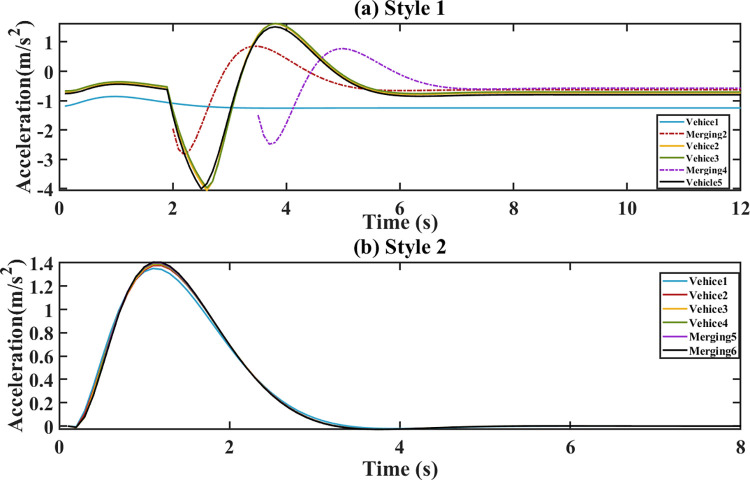
Acceleration. (a) Style 1; (b) Style 2.

**Fig 8:**
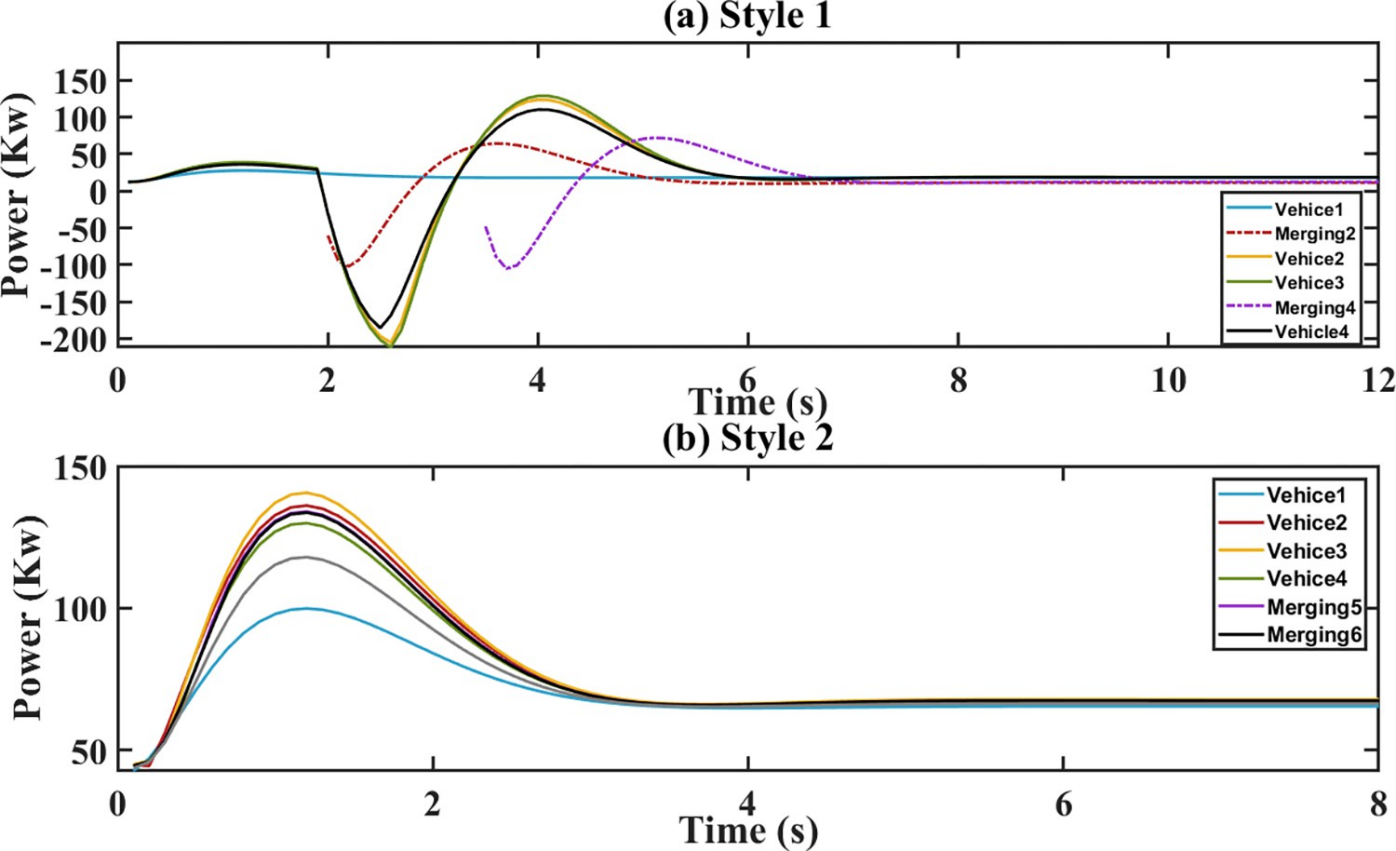
Demand power. (a) Style 1; (b) Style 2.

The platoon’s initial speed changes from 30 m/s to 32 m/s. The torque, acceleration and demand power are respectively shown in Figs 6–8. For style 1, there are sharp fluctuations of torque, acceleration and demand power among the following vehicles during the 3–5 seconds. Despite the initial fluctuations, platoon stability is quickly achieved. This indicates that the merging control strategy effectively manages the adjustments of torque, acceleration and demand power to stabilize the platoon. For stale 2, the torque, acceleration and demand power of the following vehicles respond quickly to the leader’s parameters in style 2. During the following process, they increase sharply to ensure a smaller gap. After 5 s, they tend to converge.

**Fig 9:**
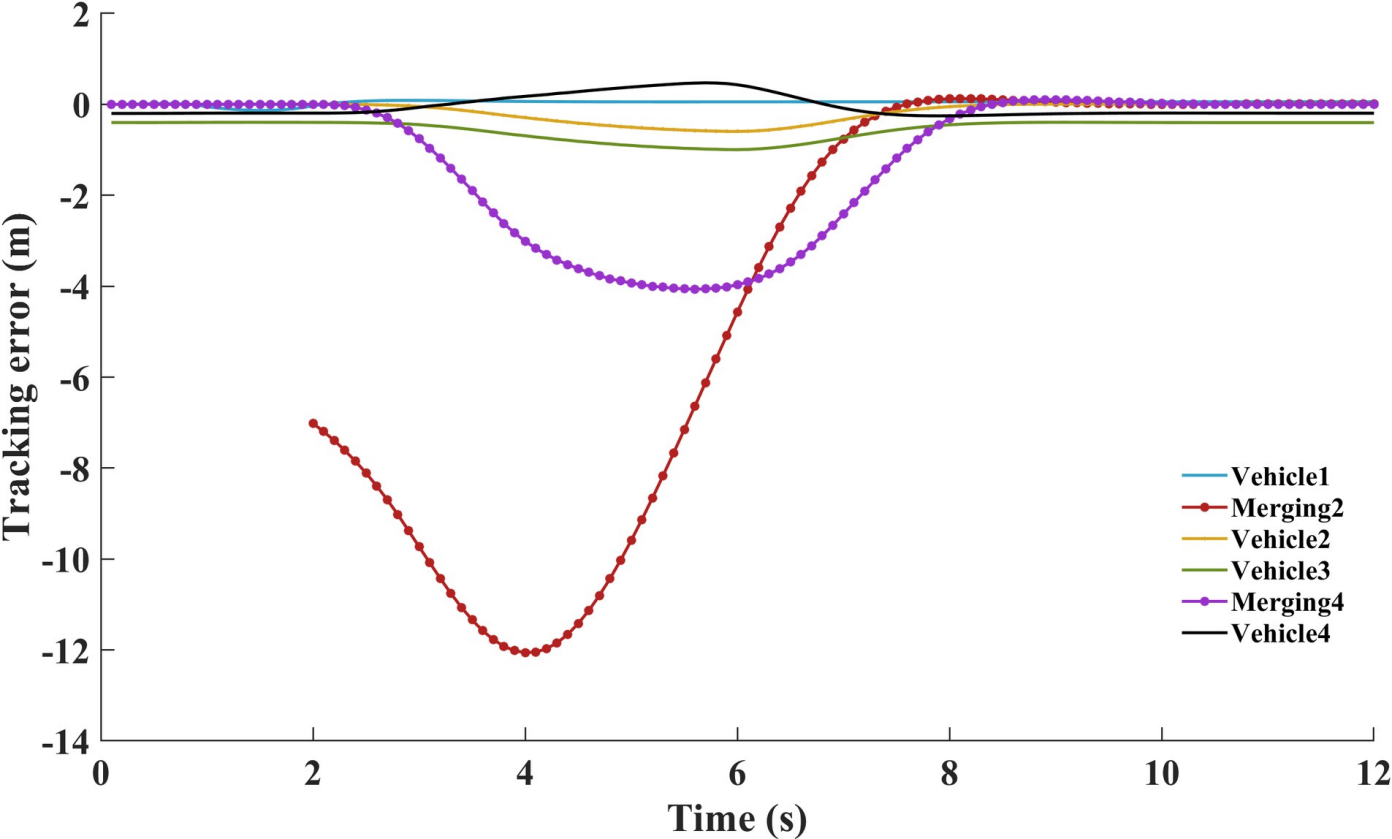
Tracking error for style 1.

The existing disruptions of ‘Merging 2’ and ‘Merging 4’ cause temporary jumping trend of the tracking error. The trend is caused by two vehicles merging task at 2-7s in Fig 9. The following vehicles respond to the merging task by increasing their velocity. The adjustment is made to maintain a stable following distance. Due to merging positions and potential disturbances in the platoon, there are errors’ accumulation of the front vehicles. However, accumulation contributes to difficulties in maintaining a small range error for ‘Merging 4’ position. By adjusting the speed, torque, acceleration and power, these accumulated errors are gradually reduced to keep platoon’s stability. Despite the initial jumping in tracking error, the tracking error tends to converge after 8 seconds. The platoon can recover and stabilize over time.

**Fig 10:**
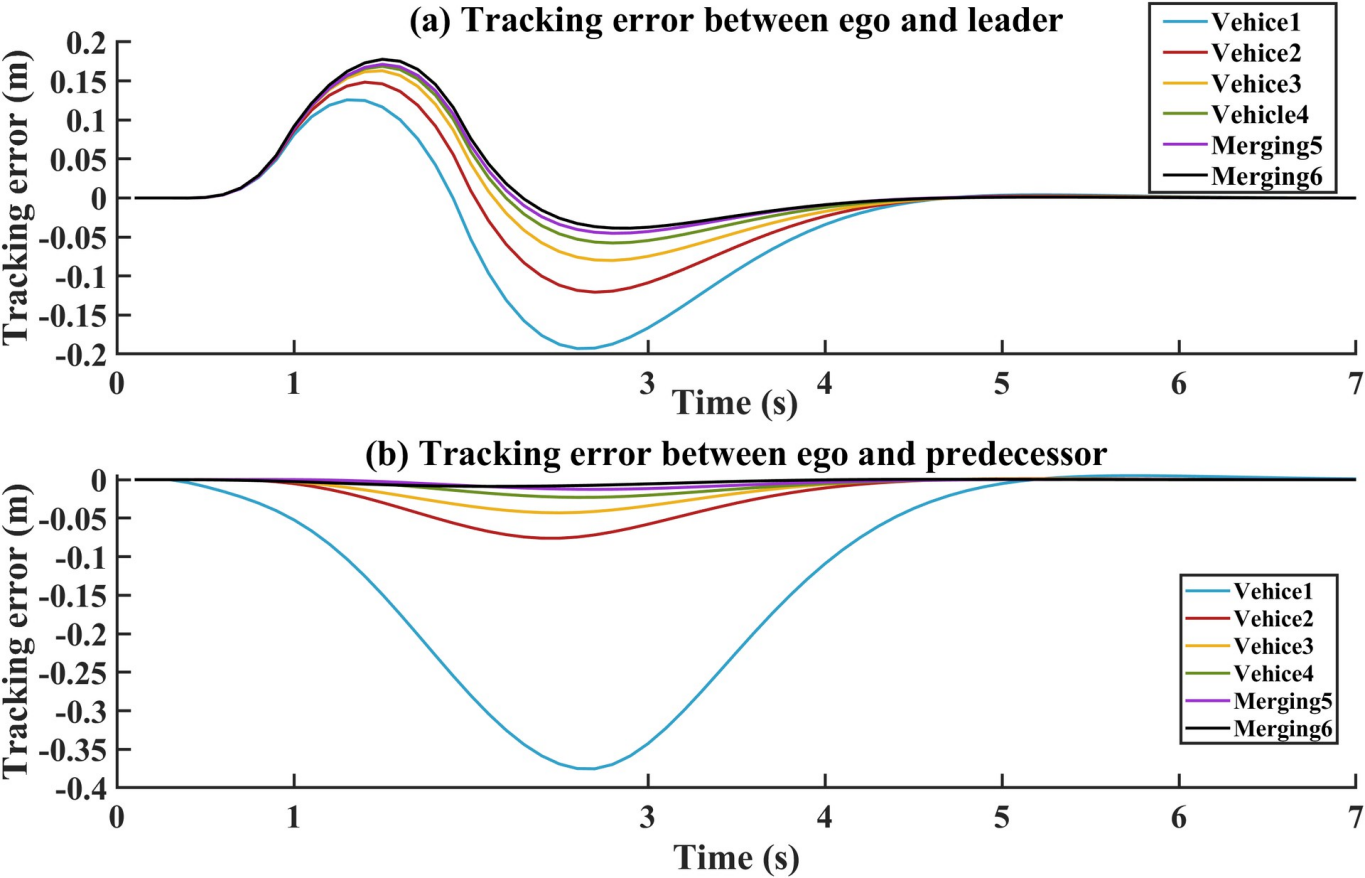
Tracking error for style 2. (a)Tracking error between the ego and leader. (b) Tracking error between the ego and followers.

According to the PFL topology, the ego maintains connections between the leader and predecessor-followers. The tracking errors can be used to evaluate the tracking performance and stability for style 2. In Fig 10(A), the curves represent the tracking distance error between the ego and the leader. In Fig 10(B), the curves represent the tracking distance error between ego and its predecessor-followers. As shown in Fig 10(A) and 10(B), the distance tracking error is effectively managed and maintained within a small range. The tracking distance error is maintained at ±0.2 m between the ego and leader, and the tracking distance error is maintained at 0.4 m between the ego and predecessor-followers. Downstream vehicles, which are relatively far away from the leader, are less likely to receive interference from random triggers. Due to the greater distance from the leader, the downstream vehicles experience a small change in tracking error. Compared with style 1, the Eco-CACC merging control method not only decreases the tracking error but also contributes to quickly achieving stability for style 2. This ensures that the tracking error fluctuates within a small range and maintains a stable tracking distance even during constant-speed driving during the merging task.

**Fig 11:**
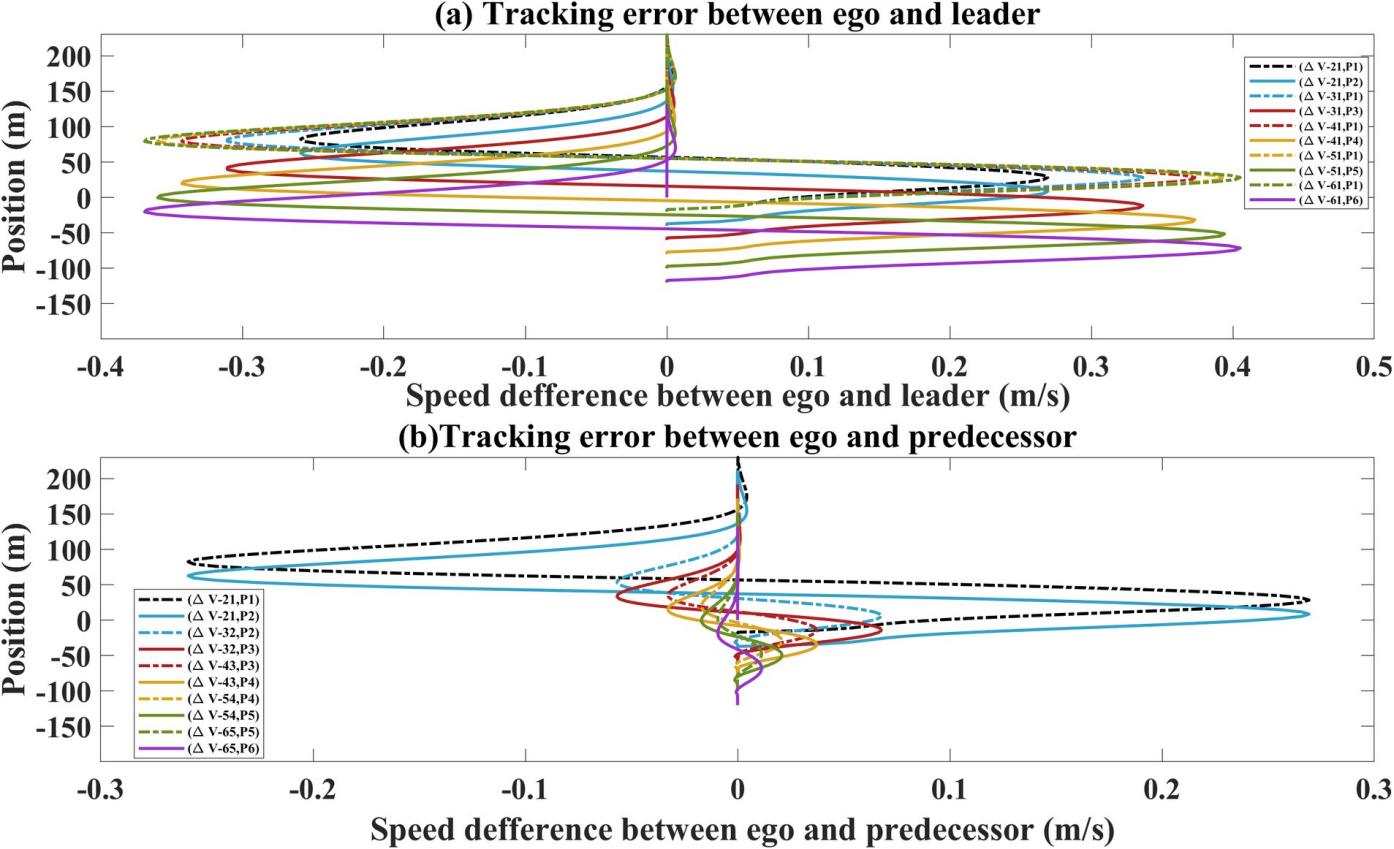
Trajectories and the speed difference for style 2. (a) between the ego and leader. (b) between the ego and predecessor-followers.

In the Fig 11(A), the dashed lines represent leader’s position determined by the speed difference between ego and leader during merging task. When downstream vehicles communicate with leader, the change are obvious in the speed difference than upstream vehicles. However, the maximum value of speed difference is 0.14 m/s. This is likely a result of reduced susceptibility to random triggers or disturbances. This is a positive outcome, as it implies a robust and consistent performance in coordination within the platoon. During the communication with leader, the speed difference is broadened due to the increase of the distance between each ego and leader. The solid lines represent the position of each ego determined by the speed difference between ego and leader. The trajectories of each ego and its predecessor-follower have a good following performance. Compared with style 1, the proposed model exhibits superior tracking stability with stable leader navigation for style 2. The Fig 11(B) shows the relationship trajectories and the speed difference. The dashed lines represent the leader’s position determined by the speed difference between ego and its predecessor. With the leader’s trajectory, the speed differences significantly decrease with each ego’s predecessors. The solid lines represent the position of each ego determined by its followers. With the leader’s trajectory, the speed differences have significantly reduction with each ego’s followers with the similar trend of dashed line. The speed difference varies less, and the individual vehicles follow well. A random disturbance occurs by merging task, the stability gradually performs well.

**Fig 12:**
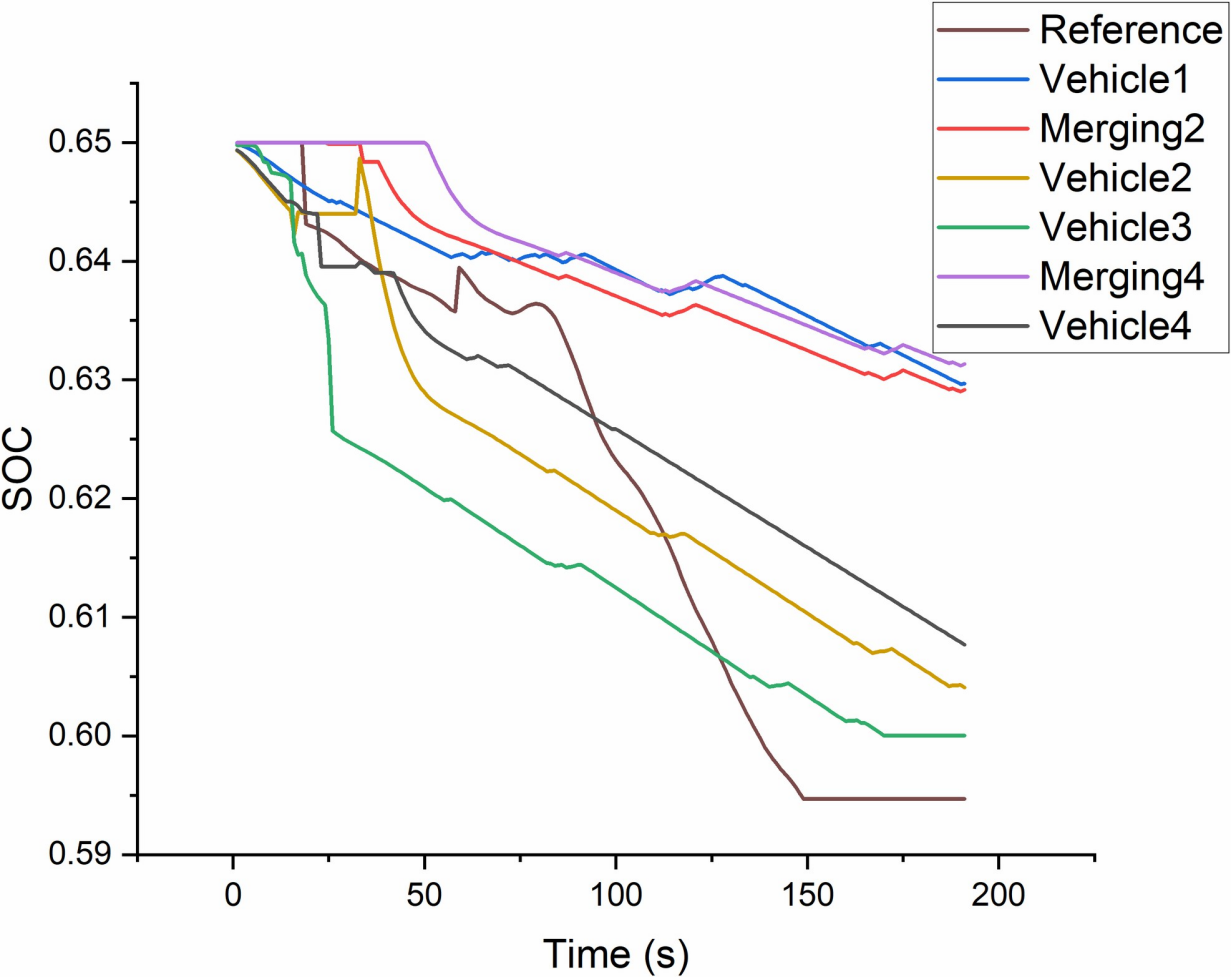
SOC curves of the platoon members for style 1.

**Fig 13:**
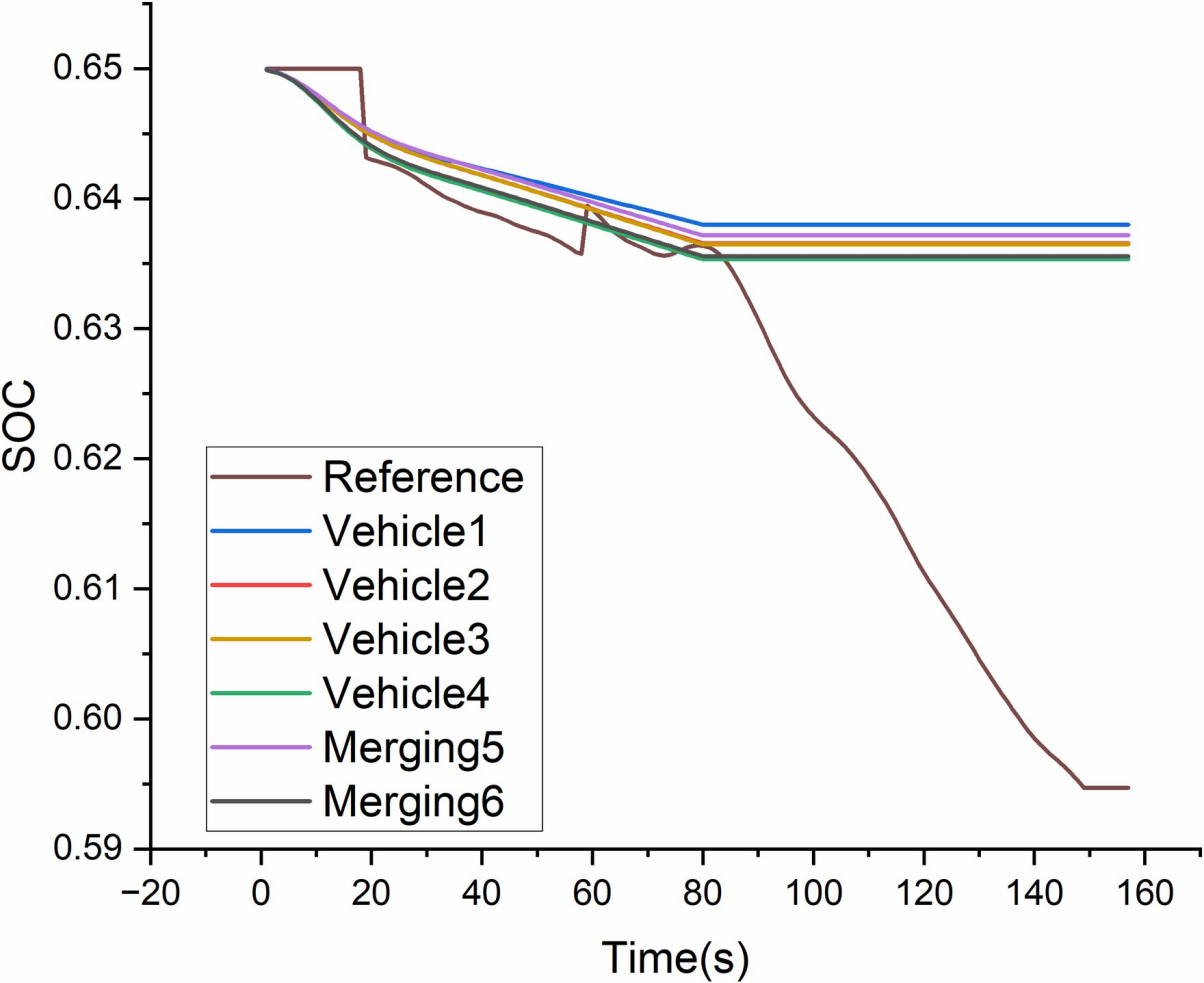
SOC curves of the platoon members for style 2.

Figs 12 and 13 depict the SOC of platoon member curves for two merging positions, which the style 2 vary within a relatively small range than Style 1. The reference SOC is evaluated by a single vehicle as the same speed cycle. The range of reference SOC is 7.6% fluctuation. Vehicles of sub-platoon are successful located at Merging 2 position and Merging 4 position as Fig 3. It shows that their SOC ranges are both less than 3% due to the eco-benefit effect on merging vehicles in the new platoon. However, SOC ranges of members in the original platoon are up to 7.8%. Fig 13 depicts the SOC of sub-platoon which is successful merged into trail position of original platoon, which ranges is less than 2.3%.

**Fig 14:**
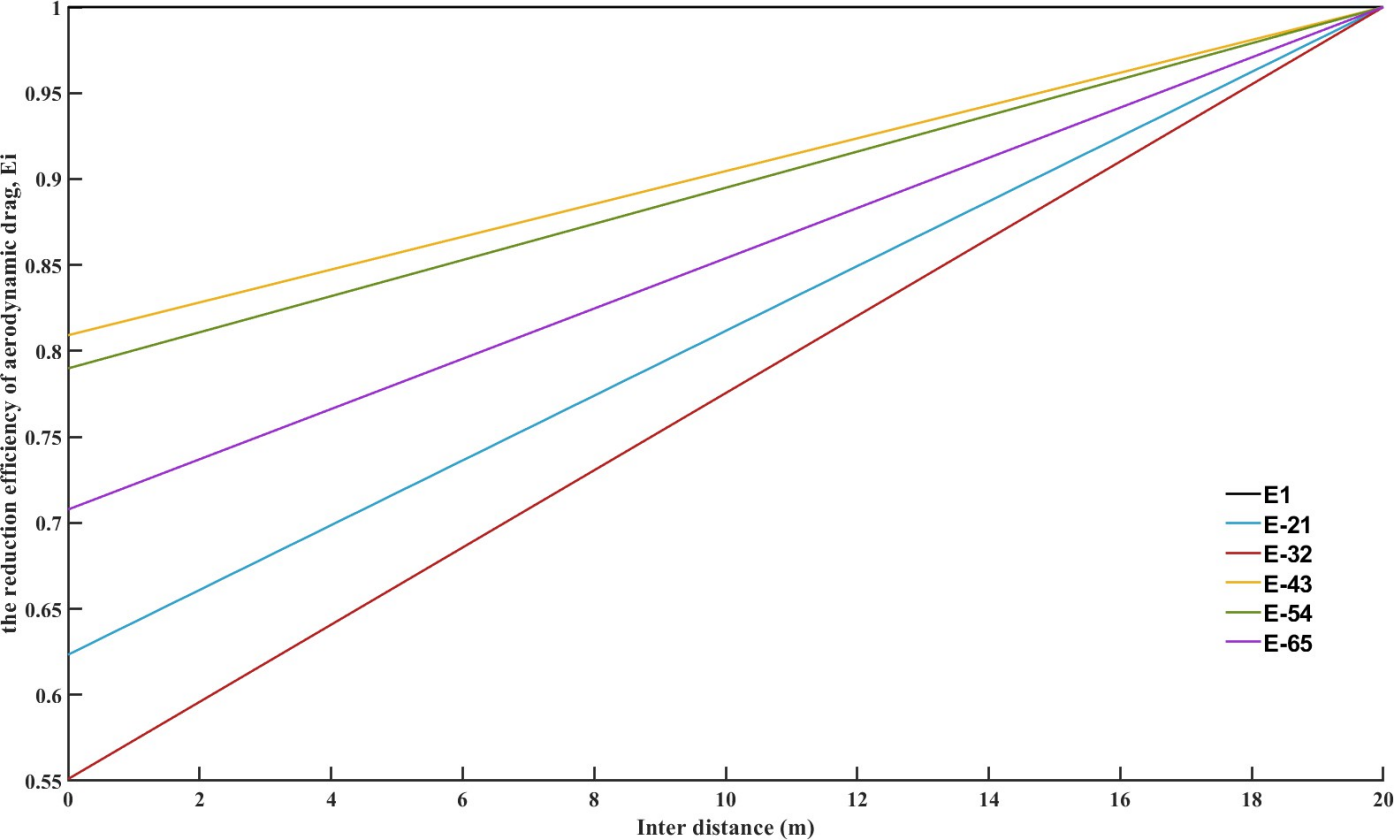
Reduction coefficient of aerodynamic drag in desired distance.

**Fig 15:**
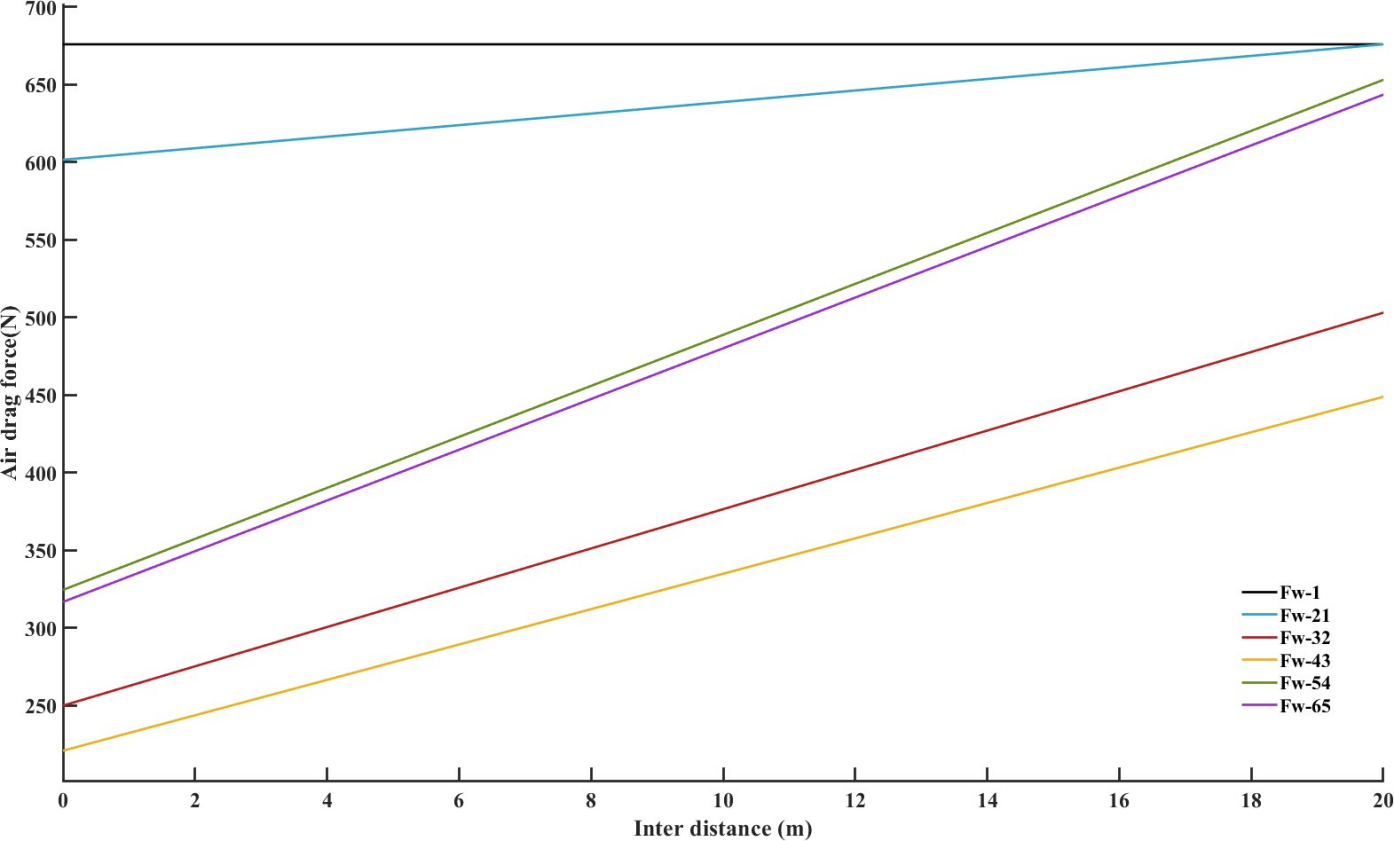
Aerodynamic drag in desired distance.

According Eq (6), the reduction coefficient of aerodynamic drag in a heterogeneous platoon after regrouping a new platoon was revised as shown in Fig 14. The black line *E*_1_ represents reference value. It is coefficient of aerodynamic drag without platooning, the value is 100%. The desire distance is 20m between inter-vehicles. The performance of two merging styles were conducted. According Eq (7), all members’ aerodynamic drags are evaluated in the regrouped platoon after merging task in Fig 15. The black line *F*_*w*1_ represents reference aerodynamic drag without platooning, the value is 684N.

The overall reduction coefficient of aerodynamic drag between leader-follower and predecessor-followers approaches that of the reference vehicle when they are in unorganized driving. This highlights the contrast between aerodynamic interactions in platoon formation and when vehicles are not closely aligned. The modified air drag coefficient increases as the inter-vehicle distance grows. It is obvious that as vehicles move farther apart, the aerodynamic resistance they face becomes more significant. The aerodynamic drag of the leader and its following vehicle is higher than that of other predecessor-followers. This indicates that the interaction between the leader and its immediate follower has a notable impact on aerodynamic drag. Generally, following vehicles have a drag reduction effect on both predecessors and the leader in the platoon. This aligns with the drafting effect, where trailing vehicles experience lower air resistance due to the leading vehicle creating a low-pressure zone. As the overall air drag coefficient between leader-follower and predecessor-followers reach to that of the reference vehicle when they are in unorganized driving.

**Fig 16:**
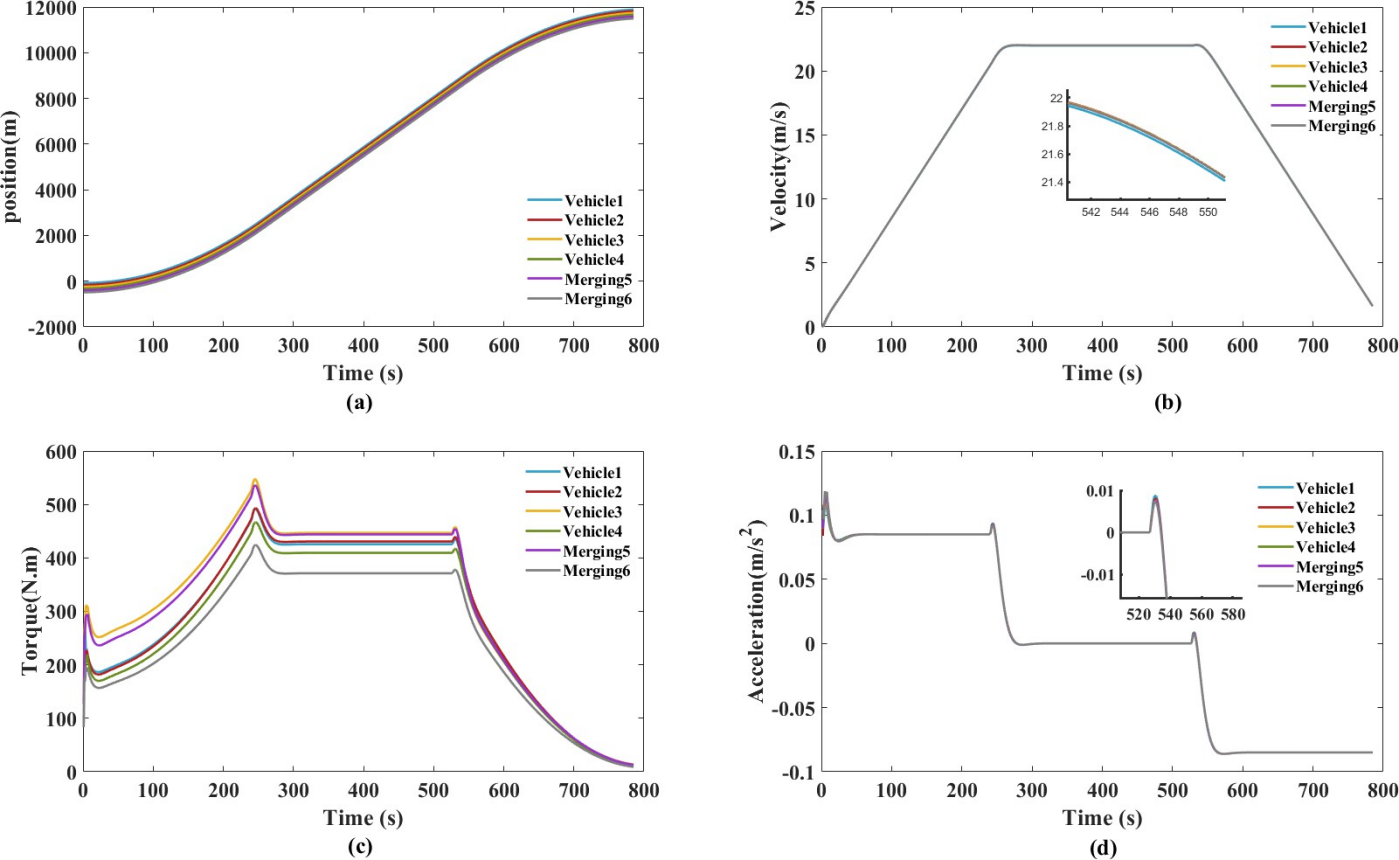
Trapezoidal velocity profile for style 1. (a) Spatiotemporal trajectory diagram. (b) Velocity. (c) Torque. (d) Acceleration.

**Fig 17:**
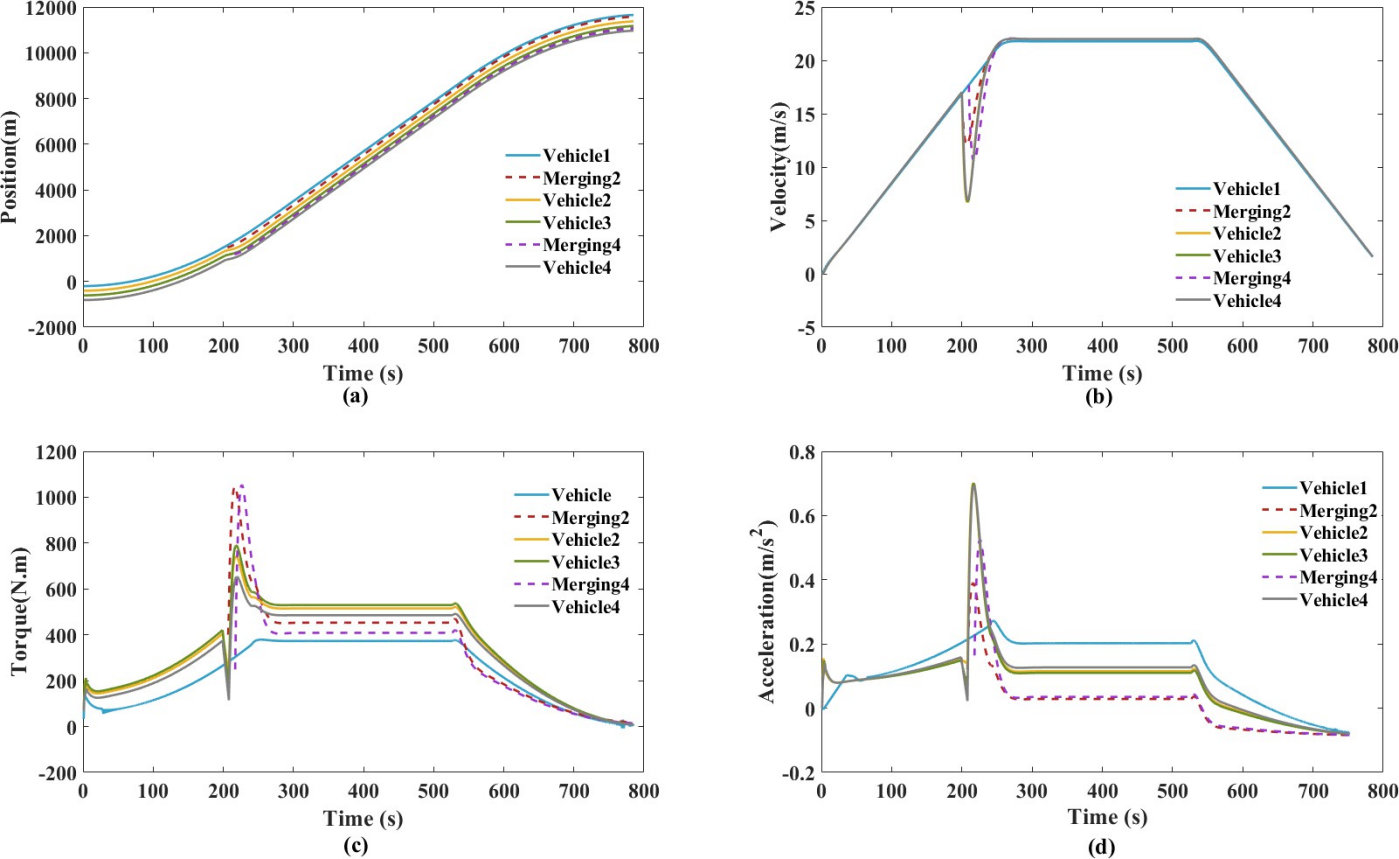
Trapezoidal velocity profile for style 2. (a) Spatiotemporal trajectory diagram. (b) Velocity. (c) Torque. (d) Acceleration.

The experiment of trapezoidal velocity profile aims to validate the simulated time responses. The maximum speed is 22m/s, two merging types are simulated by the trapezoidal velocity profile in Figs 16 and 17. The headway was measured for over 10,000 vehicles by speed, headway computation technique. During 60 to 80 km/h, the cumulative probability is set to 95%, the headway is over 12s [54]. According to the constant time gap spacing policy, we chose 12s as constant time gap.

Figs 16 and 17 show the measured spatiotemporal trajectory, velocity, torque and acceleration, which can be directly compared with the simulation results. Both style 1 and style 2, no collisions occurred in the new platoon. The proposed cooperative merging method ensure that the speed fluctuations among the following vehicles are within a small range for style 1. This approach is beneficial for maintaining smooth and coordinated motion within the platoon.

## Conclusions

Eco-CACC heterogeneous vehicle platooning for merging is an important task in advanced driver assistance systems. The ability to reconfigure vehicle platoons without collisions is a crucial aspect of autonomous driving and platooning technologies. In this paper, the DNMPC approach is developed to model the Eco-CACC merging task for a sub-platoon. We propose two merging styles for regrouped platoon models with the PLF topology. The stability performance and tracking performance can be evaluated for a regrouped platoon. The merging effects of the vehicle position, speed, torque, acceleration, demand power, tracking error and SOC are derived by the Eco-CACC merging approach. To validate the simulated time responses, we conduct spatiotemporal trajectory, velocity, torque and acceleration with trapezoidal velocity profile. Downstream vehicles are less likely to receive interference from random triggers. This is an important consideration because it affects the stability of coordination within the platoon. Compared with style 1, style 2 has superior stability performance, tracking performance and SOC when communicating with leaders and predecessor-followers. The position of the leading vehicle in the sub-platoon after it completes the merging task be as close as possible to the leading vehicle in the gap. The proposed method is adaptive and capable of handling variations in platoon speed and maintaining stable coordination among vehicles. In the future, more complex merging positions can also be analyzed in the DNMPC-STL. Furthermore, we will consider the merging behavior of multiple positions and vehicle groups in a reorganized platoon, aiming to develop a comprehensive understanding of platoon dynamics.

## Supporting information

S1 File Workspace for type 1. (MAT)

S2 File Workspace for type 2. (MAT)
